# Supplementary material for: Preclinical Evaluation of Artesunate as an Antineoplastic Agent in Ovarian Cancer Treatment
Source: Diagnostics (Basel). 2021 Feb 26;11(3):395. doi: 10.3390/diagnostics11030395 (PMC7996621; doi:10.3390/diagnostics11030395)
Supplement: Supplementary file 1 [file diagnostics-11-00395-s001.pdf]

Supplemental Table 1: Significantly differentially expressed genes between the more resistant (1226 and 1254) and sensitive (2238, 2326, 1236, 1267) organoid models. RNA extracted from untreated cells prior to RNAseq analysis

	Genes	Entrez	Symbol	logFC	logCPM	LR	PValue	FDR
4052	ENSG00000049323	4052	LTBP1	-3.712451103	6.671325178	31.67667021	1.82E-08	0.000288068
358	ENSG00000240583	358	AQP1	7.886463182	7.403485877	30.56409805	3.23E-08	0.000288068
2562	ENSG00000166206	2562	GABRB3	5.275967129	0.792020674	30.31325324	3.68E-08	0.000288068
5649	ENSG00000189056	5649	RELN	6.30372917	5.790374747	27.08301607	1.95E-07	0.001115521
393	ENSG00000089820	393	ARHGAP4	3.648923782	3.63226625	26.70299128	2.37E-07	0.001115521
100507421	ENSG00000261115	100507421	TMEM178B	5.775240781	3.091766967	25.9192505	3.56E-07	0.001394872
56106	ENSG00000253846	56106	PCDHGA10	-3.391222856	4.757983681	25.38133579	4.70E-07	0.001579965
29103	ENSG00000120675	29103	DNAJC15	-6.703255167	3.89181146	24.22450307	8.57E-07	0.002519417
4212	ENSG00000134138	4212	MEIS2	-7.982831263	4.51810315	23.66732217	1.15E-06	0.002991142
202559	ENSG00000112232	202559	KHDRBS2	5.341022621	-0.974077688	23.174086	1.48E-06	0.003478816
93035	ENSG00000205038	93035	PKHD1L1	6.58201349	7.20161776	22.7457339	1.85E-06	0.003674202
56133	ENSG00000112852	56133	PCDHB2	-5.761053448	3.122132708	22.56977922	2.03E-06	0.003674202
222611	ENSG00000164393	222611	ADGRF2	7.909995056	1.378453982	22.56477515	2.03E-06	0.003674202
26762	ENSG00000113249	26762	HAVCR1	9.059351764	1.563736678	22.21969963	2.43E-06	0.004083278
79986	ENSG00000242779	79986	ZNF702P	2.839266402	4.9803448	21.72852335	3.14E-06	0.004922571
7053	ENSG00000125780	7053	TGM3	5.607419387	3.121090963	21.30366293	3.92E-06	0.005759437
4345	ENSG00000091972	4345	CD200	3.358324198	4.086567777	19.99338286	7.77E-06	0.010746465
79627	ENSG00000119900	79627	OGFRL1	2.929478594	4.972856174	19.82696639	8.48E-06	0.011072499
64220	ENSG00000137868	64220	STRA6	-4.691119567	5.594092376	19.35699856	1.08E-05	0.013415121
646864	ENSG00000268696	646864	ZNF723	8.187126831	2.637094488	18.07553497	2.12E-05	0.024956212
64094	ENSG00000112562	64094	SMOC2	4.119487469	4.661961555	17.56100408	2.78E-05	0.031147852
6575	ENSG00000168575	6575	SLC20A2	2.421941931	6.761102839	17.44713675	2.95E-05	0.031567126
79605	ENSG00000177614	79605	PGBD5	3.170309865	4.083556611	17.11090456	3.53E-05	0.036039583
1410	ENSG00000109846	1410	CRYAB	3.040845157	6.318017736	16.79946954	4.15E-05	0.03924143
4489	ENSG00000205362	4489	MT1A	5.426336197	1.402017701	16.79102427	4.17E-05	0.03924143
1956	ENSG00000146648	1956	EGFR	1.848067746	7.695083778	16.67561678	4.43E-05	0.040098541
486	ENSG00000137731	486	FXYD2	7.540338968	1.507054672	16.16542631	5.80E-05	0.050539049
26287	ENSG00000165887	26287	ANKRD2	4.001112053	1.400305735	16.0126191	6.29E-05	0.052829502
127018	ENSG00000143353	127018	LYPLAL1	-5.864281787	2.869340203	15.13920178	9.99E-05	0.080518251
100289473	ENSG00000232528	100289473	LOC100289473	5.810391697	-3.055641582	15.07903672	0.000103102	0.080518251
1803	ENSG00000197635	1803	DPP4	2.835637015	7.09193546	15.02360317	0.000106175	0.080518251
57091	ENSG00000087589	57091	CASS4	4.086498634	0.924694953	14.53334351	0.000137701	0.100060606
9244	ENSG00000006016	9244	CRLF1	5.704817144	3.960914346	14.45112733	0.000143844	0.100060606
147372	ENSG00000183287	147372	CCBE1	5.467165888	1.238072466	14.4201925	0.000146226	0.100060606
2744	ENSG00000115419	2744	GLS	3.001400887	7.250164276	14.38518956	0.000148969	0.100060606
6542	ENSG00000003989	6542	SLC7A2	4.405085855	6.175597758	14.17499927	0.000166569	0.108774226
57214	ENSG00000103888	57214	CEMIP	-4.157310682	6.156947589	13.27332742	0.000269209	0.166979556
56136	ENSG00000239389	56136	PCDHA13	3.132173485	1.962271722	13.26847883	0.000269906	0.166979556
3990	ENSG00000166035	3990	LIPC	4.087593432	2.002570153	13.12250352	0.00029177	0.175877391
5016	ENSG00000085465	5016	OVGP1	3.910490203	2.666287122	12.80494139	0.000345705	0.199728833
79750	ENSG00000151789	79750	ZNF385D	5.509056198	1.409446087	12.74733971	0.000356516	0.199728833
152078	ENSG00000174899	152078	SLC66A1L	4.166802794	0.992198158	12.74571603	0.000356826	0.199728833
7367	ENSG00000197888	7367	UGT2B17	4.709642338	1.515606794	12.65794875	0.000373972	0.204458481
3786	ENSG00000184156	3786	KCNQ3	4.016054116	3.58463284	12.60655814	0.000384396	0.20538086
6493	ENSG00000159263	6493	SIM2	5.563077053	4.22935618	12.54858328	0.000396507	0.207143963
652	ENSG00000125378	652	BMP4	-5.002372596	6.114837955	12.38470223	0.000432866	0.221222584
54532	ENSG00000145390	54532	USP53	2.332976724	7.442718165	12.3024989	0.000452352	0.223734044
647309	ENSG00000205835	647309	GMNC	5.276502369	1.188897431	12.27153146	0.000459921	0.223734044
54346	ENSG00000112494	54346	UNC93A	-7.654829267	6.560213461	12.24570348	0.000466331	0.223734044
590	ENSG00000114200	590	BCHE	4.447055565	3.076645537	12.19905189	0.000478138	0.224810972
118663	ENSG00000138152	118663	BTBD16	4.953443867	0.836822439	12.08367235	0.000508653	0.234469129
151887	ENSG00000091986	151887	CCDC80	-3.166134415	5.136601803	12.04321876	0.000519811	0.235004712
56142	ENSG00000081842	56142	PCDHA6	-10.50994466	2.124403313	11.99819917	0.00053252	0.23620771
164832	ENSG00000170500	164832	LONRF2	3.603296693	4.97305819	11.88157906	0.000566919	0.246809048
5644	ENSG00000204983	5644	PRSS1	-5.363639716	4.639812943	11.80091001	0.000592018	0.249056795
325	ENSG00000132703	325	APCS	5.036113565	-2.730590837	11.73912824	0.000611997	0.249056795
117	ENSG00000078549	117	ADCYAP1R1	5.881772059	2.894779278	11.71196681	0.000620994	0.249056795

	Genes	Entrez	Symbol	logFC	logCPM	LR	PValue	FDR
7103	ENSG00000127324	7103	TSPAN8	-8.84406303	6.037497559	11.69683524	0.000626065	0.249056795
641700	ENSG00000249751	641700	ECSCR	-7.263452441	-0.961398442	11.67961292	0.000631887	0.249056795
144568	ENSG00000166535	144568	A2ML1	-6.403601506	3.523928184	11.65065572	0.000641799	0.249056795
54221	ENSG00000172554	54221	SNTG2	4.286520496	-1.444545248	11.63782748	0.00064624	0.249056795
22837	ENSG00000082438	22837	COBLL1	2.231456734	5.886921932	11.55797869	0.000674591	0.25578986
359710	ENSG00000186190	359710	BPIFB3	7.366341339	-1.073874459	11.48296171	0.000702371	0.262095921
10800	ENSG00000173198	10800	CYSLTR1	-7.071424877	-1.142698921	11.37492284	0.000744423	0.273447568
202134	ENSG00000182230	202134	FAM153B	3.595107274	0.968486835	11.33925268	0.000758859	0.274461628
1780	ENSG00000158560	1780	DYNC1I1	-6.217585809	2.20607203	11.18124023	0.000826285	0.294320291
286827	ENSG00000213186	286827	TRIM59	2.201359631	4.913193415	11.02025655	0.000901216	0.316219198
93166	ENSG00000061455	93166	PRDM6	4.353292948	-0.379226562	10.94814337	0.000936975	0.320386055
2037	ENSG00000079819	2037	EPB41L2	1.711309745	7.297758878	10.92706899	0.000947693	0.320386055
23236	ENSG00000182621	23236	PLCB1	1.960188979	5.057250718	10.91482731	0.000953976	0.320386055
5645	ENSG00000275896	5645	PRSS2	-5.084001225	4.6361847	10.88305389	0.000970481	0.321338552
3362	ENSG00000158748	3362	HTR6	6.36382178	-0.745938993	10.82817592	0.000999671	0.326406379
3097	ENSG00000010818	3097	HIVEP2	1.648854618	7.422487881	10.69921766	0.001071808	0.343973035
445815	ENSG00000241978	445815	PALM2AKAP2	2.05815075	7.318903681	10.65601779	0.001097134	0.343973035
55423	ENSG00000089012	55423	SIRPG	6.628780754	-1.109714786	10.65562592	0.001097366	0.343973035
6926	ENSG00000135111	6926	TBX3	-4.385386777	3.426598123	10.52225981	0.001179452	0.364838545
7373	ENSG00000187955	7373	COL14A1	3.901559436	5.850930329	10.48898072	0.001200886	0.366644541
90625	ENSG00000233056	90625	ERVH48-1	7.076401567	-1.333946695	10.42543481	0.001242916	0.374611671
83539	ENSG00000154080	83539	CHST9	3.905009122	1.465910161	10.37923337	0.001274406	0.37924064
3397	ENSG00000125968	3397	ID1	-3.987516311	5.677481583	10.23601391	0.001377249	0.404233283
7051	ENSG00000092295	7051	TGM1	3.588855418	5.370072459	10.2153292	0.001392781	0.404233283
6653	ENSG00000137642	6653	SORL1	1.838974446	8.391745243	10.15720618	0.001437383	0.41209064
9411	ENSG00000137962	9411	ARHGAP29	2.283255408	8.761416491	10.08305056	0.001496394	0.423840054
8626	ENSG00000073282	8626	TP63	4.231248802	3.353195434	10.0513416	0.00152237	0.426064338
90993	ENSG00000157613	90993	CREB3L1	-5.528615995	5.317086781	9.9961813	0.001568652	0.431324482
1180	ENSG00000188037	1180	CLCN1	-6.283895673	-1.80324992	9.985403479	0.00157786	0.431324482
51473	ENSG00000146038	51473	DCDC2	2.409616851	6.971455589	9.957044783	0.001602351	0.432984591
5579	ENSG00000166501	5579	PRKCB	2.977587166	2.210039038	9.924707998	0.001630747	0.433890765
102724594	ENSG00000275895	102724594	U2AF1L5	-7.800391759	-0.477577465	9.911360126	0.001642617	0.433890765
1510	ENSG00000196188	1510	CTSE	-8.521407042	7.775485476	9.864713258	0.001684789	0.440085693
137797	ENSG00000197353	137797	LYPD2	-7.922125421	5.90487008	9.828566205	0.001718222	0.443886694
3860	ENSG00000171401	3860	KRT13	-4.648937843	2.73573514	9.693063241	0.001849649	0.472060815
91607	ENSG00000172716	91607	SLFN11	-2.311626159	5.556216383	9.670285206	0.001872722	0.472060815
440279	ENSG00000137766	440279	UNC13C	4.999498316	0.720973726	9.644448915	0.001899247	0.472060815
51435	ENSG00000168077	51435	SCARA3	1.916606666	8.080365962	9.636387592	0.0019076	0.472060815
9965	ENSG00000162344	9965	FGF19	-9.608227106	5.017545219	9.582026561	0.001964914	0.481178782
5801	ENSG00000153233	5801	PTPRR	-7.009186372	4.36701296	9.482426464	0.002074494	0.498669721
84675	ENSG00000147573	84675	TRIM55	3.382511494	0.16318791	9.478655379	0.002078763	0.498669721
8395	ENSG00000107242	8395	PIP5K1B	-3.038009049	3.532513963	9.375004026	0.002199642	0.517952066
4982	ENSG00000164761	4982	TNFRSF11B	2.34747782	4.792448868	9.346140206	0.002234556	0.517952066
441168	ENSG00000188820	441168	CALHM6	3.936128059	-1.585284286	9.338551332	0.002243828	0.517952066
6515	ENSG00000059804	6515	SLC2A3	-6.387134795	6.026841682	9.33574125	0.002247272	0.517952066
182	ENSG00000101384	182	JAG1	1.811362163	7.375758563	9.309714932	0.002279419	0.520260892
145581	ENSG00000165379	145581	LRFN5	5.00468517	3.80180233	9.227448526	0.002384136	0.528263462
8431	ENSG00000131910	8431	NR0B2	-7.347847446	-0.878725724	9.205094401	0.002413426	0.528263462
7644	ENSG00000167232	7644	ZNF91	1.928805097	8.92177351	9.183316955	0.00244231	0.528263462
8835	ENSG00000120833	8835	SOCS2	-5.194150461	1.242396835	9.16114087	0.002472084	0.528263462
3120	ENSG00000232629	3120	HLA-DQB2	-9.606685313	1.238080754	9.158393659	0.002475798	0.528263462
3084	ENSG00000157168	3084	NRG1	3.559872315	3.810055628	9.151189439	0.002485564	0.528263462
387914	ENSG00000180730	387914	SHISA2	4.236455054	2.132733448	9.12632857	0.002519568	0.528263462
79739	ENSG00000137941	79739	TTLL7	-4.532109541	3.662872007	9.125029563	0.002521358	0.528263462
1280	ENSG00000139219	1280	COL2A1	-9.288236279	6.275452877	9.112822807	0.002538239	0.528263462
81856	ENSG00000213020	81856	ZNF611	1.76441673	6.222435048	9.112138836	0.002539188	0.528263462
92129	ENSG00000147223	92129	RIPPLY1	4.606676224	-2.085327751	9.072631269	0.002594635	0.535063706
64072	ENSG00000107736	64072	CDH23	2.669596845	2.216881911	9.029933476	0.002655941	0.535712448
118788	ENSG00000155629	118788	PIK3AP1	2.653190991	5.096523534	9.02147772	0.002668256	0.535712448
114795	ENSG00000139364	114795	TMEM132B	3.146108352	3.789393363	9.014710192	0.002678154	0.535712448

	Genes	Entrez	Symbol	logFC	logCPM	LR	PValue	FDR
26585	ENSG00000166923	26585	GREM1	-7.33721792	-0.897649707	8.977392947	0.002733404	0.535712448
92747	ENSG00000125999	92747	BPIFB1	-7.037005938	4.13290186	8.976119176	0.00273531	0.535712448
347365	ENSG00000102313	347365	ITIH6	-6.035053976	-1.992120422	8.97193723	0.002741578	0.535712448
85477	ENSG00000006747	85477	SCIN	3.748660831	2.608688202	8.955604981	0.002766196	0.535712448
64399	ENSG00000164161	64399	HHIP	-8.370624614	0.062349428	8.946458234	0.002780081	0.535712448
23742	ENSG00000185823	23742	NPAP1	5.714979757	-3.089563157	8.900854681	0.002850372	0.541306022
192668	ENSG00000205795	192668	CYS1	4.576606778	-1.584502047	8.897790324	0.00285516	0.541306022
83891	ENSG00000109762	83891	SNX25	2.164450323	5.652825078	8.826093879	0.002969533	0.558486073
284366	ENSG00000213022	284366	KLK9	4.814881807	-1.700002537	8.788088458	0.003032038	0.560974411
83988	ENSG00000104490	83988	NCALD	2.28692306	4.374078314	8.774090657	0.003055394	0.560974411
94122	ENSG00000147041	94122	SYTL5	-5.968878208	1.184744908	8.769370588	0.003063311	0.560974411
90627	ENSG00000133121	90627	STARD13	1.92308038	3.760835316	8.760520087	0.003078213	0.560974411
650368	ENSG00000223756	650368	TSSC2	3.151364003	-0.871963321	8.728267563	0.003133141	0.565277128
8854	ENSG00000128918	8854	ALDH1A2	-6.794124501	5.537601746	8.718533602	0.003149913	0.565277128
51294	ENSG00000113555	51294	PCDH12	-8.268443525	-0.03132992	8.688530785	0.003202186	0.565755398
2914	ENSG00000124493	2914	GRM4	2.980228037	-1.63815545	8.68569994	0.003207164	0.565755398
130132	ENSG00000162944	130132	RFTN2	-7.510224838	-0.747040497	8.675719876	0.003224774	0.565755398
414157	ENSG00000203942	414157	C10orf62	4.248981076	-2.766524548	8.617239544	0.003329961	0.576299403
80832	ENSG00000100336	80832	APOL4	-4.700532573	2.379254994	8.615084824	0.003333903	0.576299403
660	ENSG00000102010	660	BMX	-4.613506927	-1.392825792	8.563598818	0.003429508	0.58398516
653509	ENSG00000122852	653509	SFTPA1	-7.435305927	-0.824080367	8.555045782	0.003445658	0.58398516
79191	ENSG00000177508	79191	IRX3	3.663871643	2.871698367	8.540590481	0.003473129	0.58398516
910	ENSG00000158485	910	CD1B	5.527272457	-3.17405193	8.538181751	0.003477729	0.58398516
116328	ENSG00000165084	116328	C8orf34	3.195194371	2.847251751	8.522487886	0.003507846	0.584692515
6271	ENSG00000160678	6271	S100A1	3.447395217	6.375334954	8.505422974	0.003540896	0.584692515
1472	ENSG00000101441	1472	CST4	-7.996153438	-0.326039675	8.497394508	0.003556554	0.584692515
728229	ENSG00000278558	728229	TMEM191B	-4.308069348	-0.493250054	8.468023887	0.003614439	0.590082234
50940	ENSG00000128655	50940	PDE11A	-4.418502367	0.537290566	8.445229772	0.003660022	0.59340314
7450	ENSG00000110799	7450	VWF	-4.969857596	5.539403207	8.415783893	0.003719774	0.593448188
125893	ENSG00000180257	125893	ZNF816	1.465053909	5.305301972	8.409006659	0.003733666	0.593448188
1006	ENSG00000150394	1006	CDH8	5.42891136	-0.251864666	8.400044772	0.003752118	0.593448188
1901	ENSG00000170989	1901	S1PR1	-5.065496426	2.144264325	8.395614419	0.003761274	0.593448188
389058	ENSG00000204335	389058	SP5	-5.362631028	3.514580972	8.362618344	0.003830183	0.597960567
221395	ENSG00000069122	221395	ADGRF5	-5.13947069	1.939898733	8.35601601	0.003844125	0.597960567
978	ENSG00000158825	978	CDA	-5.744496053	2.07515998	8.34562138	0.003866179	0.597960567
6091	ENSG00000169855	6091	ROBO1	-2.117938592	6.686520395	8.316059014	0.003929609	0.601820958
101060684	ENSG00000273136	101060684	NBPF26	1.70889007	3.545757859	8.310184724	0.003942338	0.601820958
768	ENSG00000107159	768	CA9	-7.399549849	6.838051861	8.281079497	0.004006031	0.60413129
158401	ENSG00000165181	158401	SHOC1	-6.645060812	-1.52970904	8.277219398	0.004014556	0.60413129
50853	ENSG00000136059	50853	VILL	-3.494397904	6.450459422	8.26647227	0.00403839	0.60413129
8995	ENSG00000120337	8995	TNFSF18	4.963196784	-0.413404613	8.249275434	0.004076827	0.60413129
57553	ENSG00000243156	57553	MICAL3	1.485503291	6.663524494	8.230183832	0.004119936	0.60413129
2888	ENSG00000115290	2888	GRB14	3.495175402	2.508490903	8.227125006	0.004126885	0.60413129
7402	ENSG00000152818	7402	UTRN	1.499292151	8.411595538	8.218478143	0.004146596	0.60413129
869	ENSG00000102924	869	CBLN1	3.751206039	-2.178839847	8.211289582	0.004163055	0.60413129
3235	ENSG00000128709	3235	HOXD9	-5.985704823	1.909894951	8.175672603	0.004245592	0.612329021
55074	ENSG00000164830	55074	OXR1	1.509369547	6.166480103	8.116127525	0.004387318	0.617948504
6348	ENSG00000277632	6348	CCL3	3.848396037	-0.320583993	8.097874342	0.00443172	0.617948504
218	ENSG00000108602	218	ALDH3A1	-3.231039318	4.707127687	8.085638623	0.004461741	0.617948504
388559	ENSG00000213793	388559	ZNF888	2.134006207	3.142098452	8.078830494	0.004478534	0.617948504
8935	ENSG00000005020	8935	SKAP2	1.852869571	4.400043358	8.076292193	0.004484812	0.617948504
1191	ENSG00000120885	1191	CLU	2.200643216	10.59058565	8.060790315	0.004523345	0.617948504
5629	ENSG00000117707	5629	PROX1	-7.856720902	3.443617893	8.058798831	0.00452832	0.617948504
3249	ENSG00000105707	3249	HPN	3.268006801	3.325592404	8.050210487	0.004549837	0.617948504
8784	ENSG00000186891	8784	TNFRSF18	-5.711288547	2.275436619	8.049466048	0.004551707	0.617948504
9096	ENSG00000112837	9096	TBX18	-6.92593122	0.703390891	8.047269104	0.00455723	0.617948504
23254	ENSG00000189337	23254	KAZN	1.273572316	6.149720671	8.040735262	0.004573697	0.617948504
158471	ENSG00000106772	158471	PRUNE2	2.343905192	6.759457568	8.021461835	0.004622624	0.620990147
79825	ENSG00000114654	79825	EFCC1	-6.157537913	-1.89767705	7.998371606	0.004681944	0.625385304
116441	ENSG00000163762	116441	TM4SF18	4.315615253	1.003027572	7.964305854	0.004770878	0.63031996

	Genes	Entrez	Symbol	logFC	logCPM	LR	PValue	FDR
153328	ENSG00000145832	153328	SLC25A48	3.60579065	-1.03128412	7.963686684	0.004772511	0.63031996
6403	ENSG00000174175	6403	SELP	-10.90735371	2.520719707	7.926319132	0.004872081	0.639875723
1291	ENSG00000142156	1291	COL6A1	-2.243152192	7.905985832	7.90810511	0.004921379	0.642759475
7857	ENSG00000171951	7857	SCG2	-4.243762479	1.027629569	7.884865385	0.004985019	0.64747408
24	ENSG00000198691	24	ABCA4	3.002518566	3.878536793	7.873232399	0.005017189	0.648004035
255349	ENSG00000206069	255349	TMEM211	-6.501382234	-1.611573808	7.863513712	0.005044227	0.648004035
283659	ENSG00000166450	283659	PRTG	3.128661475	2.492422176	7.830792061	0.005136359	0.652115424
6492	ENSG00000112246	6492	SIM1	4.595142181	0.025454397	7.817820342	0.005173355	0.652115424
55600	ENSG00000179914	55600	ITLN1	-5.470762233	-2.401429454	7.814997766	0.005181441	0.652115424
1543	ENSG00000140465	1543	CYP1A1	-5.061908825	3.027238011	7.812994766	0.005187187	0.652115424
5457	ENSG00000152192	5457	POU4F1	-8.335922329	0.017236466	7.783056975	0.005273848	0.655219244
9075	ENSG00000165376	9075	CLDN2	-8.969590749	2.695952584	7.781038325	0.005279744	0.655219244
5080	ENSG00000007372	5080	PAX6	3.657405554	-0.553924702	7.76100372	0.005338628	0.655219244
147920	ENSG00000204866	147920	IGFL2	-6.355451221	-1.772483716	7.754382922	0.005358235	0.655219244
57611	ENSG00000167178	57611	ISLR2	-5.371956805	0.139800465	7.744765829	0.005386845	0.655219244
25928	ENSG00000171243	25928	SOSTDC1	3.561469831	4.652042333	7.741757725	0.005395825	0.655219244
11145	ENSG00000176485	11145	PLAAT3	-2.047025492	4.760186178	7.738030824	0.005406973	0.655219244
338557	ENSG00000186188	338557	FFAR4	-8.391510689	0.086299845	7.699431568	0.005523822	0.665946299
79574	ENSG00000198758	79574	EPS8L3	-7.915488378	4.489878111	7.689216384	0.005555174	0.666309139
23322	ENSG00000103494	23322	RPGRIP1L	1.684705138	4.211538869	7.663526705	0.005634825	0.669451588
5672	ENSG00000243137	5672	PSG4	4.547152869	0.418665838	7.659041715	0.00564885	0.669451588
55103	ENSG00000116191	55103	RALGPS2	1.448140802	5.25028171	7.653317131	0.005666803	0.669451588
5608	ENSG00000108984	5608	MAP2K6	-1.576739642	5.00914885	7.640412998	0.005707485	0.670886299
301	ENSG00000135046	301	ANXA1	-1.787071547	8.549408444	7.625041872	0.005756333	0.672589859
4842	ENSG00000089250	4842	NOS1	4.545598111	4.473361171	7.617892906	0.005779197	0.672589859
155465	ENSG00000173467	155465	AGR3	-3.6454203	4.105961902	7.578093676	0.005908189	0.682618998
163747	ENSG00000162398	163747	LEXM	-4.274105093	-1.673456784	7.572471365	0.005926647	0.682618998
4879	ENSG00000120937	4879	NPPB	-10.75129254	2.348953895	7.564631678	0.005952482	0.682618998
79689	ENSG00000127954	79689	STEAP4	-6.131255825	3.599636485	7.532840312	0.006058432	0.691396471
80243	ENSG00000046889	80243	PREX2	2.335151707	6.616452133	7.498900178	0.006173668	0.698538564
5874	ENSG00000041353	5874	RAB27B	-2.955401421	3.940241249	7.488604505	0.006209066	0.698538564
51268	ENSG00000179761	51268	PIPOX	3.15573808	1.342883634	7.482539336	0.006230015	0.698538564
56961	ENSG00000105251	56961	SHD	3.41772811	-2.572879336	7.479693356	0.00623987	0.698538564
7043	ENSG00000119699	7043	TGFB3	-2.648565047	3.192554608	7.462053717	0.006301308	0.702073185
10911	ENSG00000049247	10911	UTS2	-8.161716703	-0.162733263	7.361901764	0.006662029	0.735585845
23259	ENSG00000085788	23259	DDHD2	1.541404103	5.566686701	7.361188482	0.006664672	0.735585845
23670	ENSG00000135048	23670	CEMIP2	-2.262972952	7.49803409	7.336467098	0.006756951	0.740511179
643355	ENSG00000231437	643355	LINC01750	4.911638755	-3.41199991	7.332389616	0.006772296	0.740511179
11254	ENSG00000268104	11254	SLC6A14	-7.161021502	6.182705918	7.296515771	0.006908846	0.750550236
7314	ENSG00000170315	7314	UBB	-1.796962813	10.15939615	7.273719385	0.006997076	0.750550236
10595	ENSG00000134398	10595	ERN2	-7.138026976	4.606466885	7.273260102	0.006998865	0.750550236
7364	ENSG00000171234	7364	UGT2B7	3.345874854	6.159292985	7.265330359	0.007029834	0.750550236
284252	ENSG00000134504	284252	KCTD1	1.463772581	5.717960912	7.255928647	0.007066732	0.750550236
7122	ENSG00000184113	7122	CLDN5	-6.755089416	-1.379349688	7.252649807	0.007079647	0.750550236
445329	ENSG00000213648	445329	SULT1A4	-5.678749692	-2.280300204	7.250636131	0.00708759	0.750550236
9956	ENSG00000122254	9956	HS3ST2	4.45835115	-1.891031198	7.216896483	0.007222047	0.759541204
7306	ENSG00000107165	7306	TYRP1	-5.639550423	-2.287429274	7.206652654	0.007263383	0.759541204
55753	ENSG00000197444	55753	OGDHL	1.984749358	5.33696406	7.196726754	0.007303668	0.759541204
134285	ENSG00000157111	134285	TMEM171	3.026271244	2.060787282	7.193661173	0.007316156	0.759541204
80014	ENSG00000151718	80014	WWC2	1.462805912	6.147087419	7.169801234	0.007414099	0.759541204
933	ENSG00000012124	933	CD22	3.605070804	1.851589134	7.158165245	0.007462349	0.759541204
50506	ENSG00000140279	50506	DUOX2	-8.494230226	7.232646152	7.14735278	0.007507472	0.759541204
28954	ENSG00000088320	28954	REM1	4.604299806	1.12680701	7.140010126	0.007538273	0.759541204
10451	ENSG00000134215	10451	VAV3	2.753431841	4.010272516	7.131227894	0.007575283	0.759541204
911	ENSG00000158481	911	CD1C	4.815746224	-2.523924021	7.122077908	0.007614041	0.759541204
2028	ENSG00000138792	2028	ENPEP	-4.703243754	0.847694663	7.11973066	0.007624016	0.759541204
653390	ENSG00000103472	653390	RRN3P2	2.715378084	-1.621724571	7.104585494	0.0076887	0.759541204
3549	ENSG00000163501	3549	IHH	-7.738216982	-0.605247253	7.102994329	0.007695529	0.759541204
1268	ENSG00000118432	1268	CNR1	-5.008264834	-2.700785238	7.100660616	0.007705555	0.759541204
6296	ENSG00000005187	6296	ACSM3	1.942828695	4.271308894	7.097730778	0.007718161	0.759541204

	Genes	Entrez	Symbol	logFC	logCPM	LR	PValue	FDR
90139	ENSG00000157570	90139	TSPAN18	-3.19214957	1.63727268	7.097067831	0.007721016	0.759541204
405753	ENSG00000140274	405753	DUOXA2	-11.54394892	5.411182209	7.096900151	0.007721738	0.759541204
2313	ENSG00000151702	2313	FLI1	-5.804940858	3.62856378	7.081460842	0.007788549	0.759994524
54550	ENSG00000103154	54550	NECAB2	-6.522648867	-1.605097463	7.077375938	0.007806324	0.759994524
147111	ENSG00000185269	147111	NOTUM	-8.352309382	5.098693783	7.07347669	0.00782333	0.759994524
153	ENSG00000043591	153	ADRB1	-5.745213576	-0.303249342	7.047137367	0.007939203	0.765238818
10365	ENSG00000127528	10365	KLF2	-3.981401624	1.290351024	7.046412504	0.007942417	0.765238818
725	ENSG00000123843	725	C4BPB	-5.453473777	0.083783459	7.028897981	0.008020469	0.769604965
153918	ENSG00000118491	153918	ZC2HC1B	4.160178342	-3.156889257	7.015986378	0.008078512	0.770993968
5053	ENSG00000171759	5053	PAH	4.749267329	-3.46514585	7.010790316	0.008101991	0.770993968
256435	ENSG00000184005	256435	ST6GALNAC3	-4.153203071	1.736946276	7.000616534	0.008148165	0.770993968
4099	ENSG00000105695	4099	MAG	4.507178861	-0.062775918	6.996674577	0.008166128	0.770993968
342527	ENSG00000188176	342527	SMTNL2	3.220737503	0.658982458	6.983637068	0.008225827	0.773523875
4254	ENSG00000049130	4254	KITLG	-3.322658117	4.29320759	6.951243515	0.008376099	0.779510096
1109	ENSG00000198610	1109	AKR1C4	-6.279795034	-1.769019568	6.948806273	0.008387518	0.779510096
140886	ENSG00000174740	140886	PABPC5	3.008753204	-1.457970901	6.948498862	0.00838896	0.779510096
51537	ENSG00000242114	51537	MTFP1	-2.416176845	4.663309316	6.934716697	0.008453845	0.782446644
9496	ENSG00000121075	9496	TBX4	-7.028147524	-1.198847662	6.908492075	0.008578731	0.790891711
255877	ENSG00000161940	255877	BCL6B	-5.696031849	0.395647507	6.897100038	0.008633568	0.790960933
6484	ENSG00000110080	6484	ST3GAL4	-2.416941241	4.943989731	6.894368169	0.008646772	0.790960933
4744	ENSG00000100285	4744	NEFH	-10.03618322	5.624955569	6.879304042	0.008719951	0.7945633
8564	ENSG00000117009	8564	KMO	3.290748205	-0.022613389	6.848844852	0.00886986	0.797193713
26191	ENSG00000134242	26191	PTPN22	-5.855517878	2.808317149	6.847343078	0.008877319	0.797193713
10750	ENSG00000154016	10750	GRAP	-5.032755098	-1.042061888	6.84209885	0.008903417	0.797193713
333	ENSG00000105290	333	APLP1	-2.668973866	6.448696508	6.841988403	0.008903968	0.797193713
10170	ENSG00000073737	10170	DHRS9	-5.424637192	3.490941054	6.827839584	0.008974776	0.797193713
4038	ENSG00000134569	4038	LRP4	-4.100884433	6.268799353	6.823061006	0.00899882	0.797193713
5934	ENSG00000103479	5934	RBL2	1.308609167	7.178176733	6.822707367	0.009000602	0.797193713
64101	ENSG00000128594	64101	LRRC4	-3.383227026	5.849298766	6.815240801	0.009038312	0.797193713
3293	ENSG00000130948	3293	HSD17B3	-5.557145151	-2.338419241	6.809455116	0.009067643	0.797193713
25780	ENSG00000152689	25780	RASGRP3	-3.419270353	1.663873184	6.805466607	0.00908792	0.797193713
65061	ENSG00000138395	65061	CDK15	-7.993291703	-0.299426782	6.784529682	0.009195125	0.803599265
6035	ENSG00000129538	6035	RNASE1	-5.313451575	6.854810081	6.769285433	0.009273996	0.807490261
8794	ENSG00000173535	8794	TNFRSF10C	-6.088540045	3.384250445	6.741817111	0.009417865	0.816553564
10752	ENSG00000134121	10752	CHL1	3.562326455	5.398844731	6.736202032	0.009447555	0.816553564
6665	ENSG00000129194	6665	SOX15	3.63435891	-1.215181344	6.718778072	0.009540297	0.821548866
284654	ENSG00000169218	284654	RSPO1	5.380924595	0.25856593	6.708721366	0.009594249	0.823179598
57540	ENSG00000204624	57540	DISP3	-3.540834858	-1.077553877	6.67727517	0.009764979	0.825229917
7576	ENSG00000198538	7576	ZNF28	1.378626683	5.730027963	6.672239015	0.009792609	0.825229917
221833	ENSG00000164651	221833	SP8	5.390616274	-1.213290846	6.668832676	0.009811343	0.825229917
162967	ENSG00000182986	162967	ZNF320	1.566756588	4.866343435	6.656720822	0.009878253	0.825229917
80256	ENSG00000005238	80256	FAM214B	-1.803978447	4.003692083	6.654262744	0.009891889	0.825229917
730249	ENSG00000102794	730249	ACOD1	4.566264881	-3.520509766	6.646680983	0.009934071	0.825229917
401992	ENSG00000196240	401992	OR2T2	4.566264881	-3.520509766	6.646680983	0.009934071	0.825229917
150160	ENSG00000198445	150160	CCT8L2	4.566264881	-3.520509766	6.646680983	0.009934071	0.825229917
377630	ENSG00000223443	377630	USP17L2	4.566264881	-3.520509766	6.646680983	0.009934071	0.825229917
5157	ENSG00000104213	5157	PDGFRL	2.379044859	2.612533316	6.639921712	0.009971832	0.825449965
441234	ENSG00000182111	441234	ZNF716	-8.13975643	-0.165043741	6.617169649	0.010100022	0.831136204
4500	ENSG00000260549	4500	MT1L	2.770331032	2.875783311	6.610864552	0.010135844	0.831136204
390874	ENSG00000205922	390874	ONECUT3	-8.790100145	0.438504958	6.598820567	0.010204634	0.831136204
430	ENSG00000183734	430	ASCL2	-6.62732597	-1.556179052	6.596867615	0.010215834	0.831136204
928	ENSG00000010278	928	CD9	-1.782970957	8.572407994	6.596613151	0.010217294	0.831136204
5368	ENSG00000168081	5368	PNOC	3.734350303	3.658358219	6.559086116	0.010434994	0.840695593
5584	ENSG00000163558	5584	PRKCI	1.37170466	7.462453143	6.558447353	0.010438741	0.840695593
92235	ENSG00000198842	92235	DUSP27	-6.185990394	1.687270639	6.554105381	0.010464244	0.840695593
3171	ENSG00000170608	3171	FOXA3	-6.119720409	2.34035878	6.54745376	0.010503437	0.840695593
128646	ENSG00000125900	128646	SIRPD	3.247886413	-2.543683412	6.544009416	0.010523791	0.840695593
9560	ENSG00000276070	9560	CCL4L2	4.3160895	-2.428622792	6.535401839	0.010574833	0.840695593
6439	ENSG00000168878	6439	SFTPB	-5.126604019	0.263371689	6.5336701	0.010585133	0.840695593
64651	ENSG00000144655	64651	CSRNP1	-1.870237998	5.391375329	6.521518426	0.010657697	0.843608766

	Genes	Entrez	Symbol	logFC	logCPM	LR	PValue	FDR
4010	ENSG00000136944	4010	LMX1B	-7.120905235	-1.093526232	6.481320194	0.010901398	0.856051645
1307	ENSG00000084636	1307	COL16A1	-1.978937582	3.819277954	6.479608669	0.010911901	0.856051645
26	ENSG00000002726	26	AOC1	-5.364102801	5.686686819	6.477616919	0.010924135	0.856051645
158046	ENSG00000130045	158046	NXNL2	2.731371054	2.069636128	6.460786482	0.011028082	0.859161682
285025	ENSG00000163492	285025	CCDC141	-4.741749885	-2.865991563	6.459363907	0.011036915	0.859161682
10100	ENSG00000134198	10100	TSPAN2	-2.82283313	2.367947641	6.444823978	0.011127607	0.860731349
10725	ENSG00000102908	10725	NFAT5	1.478151301	7.668138308	6.444393315	0.011130305	0.860731349
80227	ENSG00000175575	80227	PAAF1	1.400385398	4.641384562	6.427054896	0.01123948	0.866324341
7980	ENSG00000105825	7980	TFPI2	-3.365833134	6.976801439	6.415387827	0.011313562	0.86755765
150	ENSG00000150594	150	ADRA2A	-5.710326096	3.552154967	6.412921418	0.011329287	0.86755765
10699	ENSG00000145244	10699	CORIN	-7.27661671	3.48091429	6.395117848	0.011443463	0.870725486
728194	ENSG00000169402	728194	RSPH10B2	3.265099454	-1.721540277	6.394921245	0.011444731	0.870725486
9945	ENSG00000131459	9945	GFPT2	-3.046433216	2.519639004	6.374821375	0.011575069	0.877800973
25981	ENSG00000114841	25981	DNAH1	1.458494612	4.422964204	6.363175709	0.011651282	0.88073953
1030	ENSG00000147883	1030	CDKN2B	-3.484583073	3.375556711	6.347219822	0.01175654	0.881435134
79639	ENSG00000126106	79639	TMEM53	-2.013084096	3.322251509	6.346081223	0.011764088	0.881435134
26281	ENSG00000078579	26281	FGF20	-8.925804628	0.536684797	6.343527093	0.011781039	0.881435134
8000	ENSG00000167653	8000	PSCA	-6.267692387	1.477259721	6.333320139	0.011849029	0.881435134
432	ENSG00000141505	432	ASGR1	-4.314462689	0.200744673	6.328277052	0.01188277	0.881435134
2199	ENSG00000163520	2199	FBLN2	-3.916631803	6.1209292	6.327877856	0.011885445	0.881435134
199777	ENSG00000188171	199777	ZNF626	2.031459727	5.513260586	6.306230356	0.012031439	0.888775868
199699	ENSG00000179284	199699	DAND5	-5.24729209	-2.556205922	6.295150961	0.012106871	0.888775868
56667	ENSG00000173702	56667	MUC13	-5.502682504	2.463103309	6.282352033	0.012194615	0.888775868
123041	ENSG00000140090	123041	SLC24A4	4.461500663	-1.308612772	6.275837359	0.012239527	0.888775868
1749	ENSG00000105880	1749	DLX5	-6.758186546	2.025556791	6.275071842	0.012244816	0.888775868
2877	ENSG00000176153	2877	GPX2	-5.909309363	4.73867452	6.274674009	0.012247565	0.888775868
401546	ENSG00000188959	401546	C9orf152	-4.571359294	3.408412637	6.269718975	0.012281862	0.888775868
3772	ENSG00000157551	3772	KCNJ15	3.415095575	0.60110803	6.263673382	0.012323841	0.888775868
285596	ENSG00000170074	285596	FAM153A	3.772294702	-0.23500598	6.263552624	0.012324681	0.888775868
5328	ENSG00000122861	5328	PLAU	2.64847968	7.405205518	6.241416939	0.012479659	0.894577725
154	ENSG00000169252	154	ADRB2	-3.510366982	3.192425074	6.231162233	0.012552133	0.894577725
503542	ENSG00000203772	503542	SPRN	4.356597292	-3.578290655	6.227639265	0.01257713	0.894577725
100996648	ENSG00000215029	100996648	TCP11X2	4.356597292	-3.578290655	6.227639265	0.01257713	0.894577725
6423	ENSG00000145423	6423	SFRP2	-5.065991163	1.062043761	6.219010632	0.012638572	0.894577725
9076	ENSG00000163347	9076	CLDN1	2.269178217	8.450033602	6.210920444	0.01269646	0.894577725
7499	ENSG00000124343	7499	XG	-5.579058241	0.743646993	6.210401296	0.012700184	0.894577725
8324	ENSG00000155760	8324	FZD7	-1.730453795	4.31674874	6.209095362	0.012709556	0.894577725
4978	ENSG00000183715	4978	OPCML	4.943809062	-2.810305088	6.201228601	0.012766167	0.89588004
6286	ENSG00000163993	6286	S100P	-5.019564266	2.369287138	6.185903271	0.012877195	0.900982104
54959	ENSG00000109205	54959	ODAM	-9.901419792	1.505682839	6.168250564	0.013006316	0.907315997
577	ENSG00000135298	577	ADGRB3	3.15439067	-0.806427218	6.152192857	0.013124927	0.912881353
23348	ENSG00000088387	23348	DOCK9	1.165470599	7.781585554	6.121524693	0.013354557	0.922594167
11005	ENSG00000133710	11005	SPINK5	-4.489785973	1.926418634	6.119137348	0.013372604	0.922594167
7135	ENSG00000159173	7135	TNNI1	-5.711047929	-2.212035824	6.117855522	0.013382305	0.922594167
8553	ENSG00000134107	8553	BHLHE40	-3.429612397	8.892218742	6.09087703	0.013588162	0.931220343
91851	ENSG00000101938	91851	CHRDL1	3.727585253	0.640961576	6.088254667	0.013608345	0.931220343
51761	ENSG00000132932	51761	ATP8A2	3.367669304	-0.078598328	6.082770578	0.013650652	0.931220343
150763	ENSG00000186281	150763	GPAT2	-5.511471014	-2.367817222	6.073156637	0.013725146	0.931220343
4502	ENSG00000125148	4502	MT2A	2.003139446	8.189007545	6.068986813	0.013757586	0.931220343
9355	ENSG00000106689	9355	LHX2	-8.029894108	1.912613529	6.05797184	0.013843658	0.931220343
284110	ENSG00000167914	284110	GSDMA	-5.845801084	-2.189602496	6.045036138	0.013945448	0.931220343
420	ENSG00000111339	420	ART4	4.038851291	-3.664408135	6.038759811	0.013995112	0.931220343
341152	ENSG00000171561	341152	OR2AT4	4.038851291	-3.664408135	6.038759811	0.013995112	0.931220343
23017	ENSG00000135472	23017	FAIM2	-3.600115651	0.612360491	6.029961263	0.014065042	0.931220343
64600	ENSG00000158786	64600	PLA2G2F	-5.630480997	-2.321342231	6.028162146	0.014079385	0.931220343
8778	ENSG00000105501	8778	SIGLEC5	4.540055928	-3.097474958	6.01790859	0.014161419	0.931220343
1401	ENSG00000132693	1401	CRP	4.353982376	-3.59322786	5.998268598	0.014319925	0.931220343
2900	ENSG00000149403	2900	GRIK4	-4.77453195	-0.013004209	5.997587526	0.014325454	0.931220343
3670	ENSG00000016082	3670	ISL1	-6.766613371	-1.474296756	5.989323654	0.01439272	0.931220343
5794	ENSG00000080031	5794	PTPRH	-4.596128886	5.540697692	5.984749807	0.014430089	0.931220343

	Genes	Entrez	Symbol	logFC	logCPM	LR	PValue	FDR
1043	ENSG00000169442	1043	CD52	-7.53626062	-0.720286971	5.97864953	0.014480086	0.931220343
6690	ENSG00000164266	6690	SPINK1	-8.904407493	4.164336556	5.972059192	0.014534299	0.931220343
7378	ENSG00000183696	7378	UPP1	-2.036744805	3.826537991	5.971066122	0.014542486	0.931220343
79623	ENSG00000158089	79623	GALNT14	1.79375746	4.154171105	5.970961128	0.014543352	0.931220343
51046	ENSG00000177511	51046	ST8SIA3	-8.614687282	0.283469545	5.970548523	0.014546755	0.931220343
279	ENSG00000243480	279	AMY2A	4.463173335	-2.623370395	5.967533452	0.014571649	0.931220343
23051	ENSG00000174306	23051	ZHX3	1.611374432	6.051516778	5.966356348	0.01458138	0.931220343
10008	ENSG00000175538	10008	KCNE3	-2.992374957	2.465299009	5.962367898	0.014614401	0.931220343
3690	ENSG00000259207	3690	ITGB3	2.074698602	5.891751117	5.960667993	0.014628498	0.931220343
2791	ENSG00000127920	2791	GNG11	-4.227507247	1.314496884	5.956565818	0.014662574	0.931220343
4973	ENSG00000173391	4973	OLR1	-5.226085128	-2.562260833	5.955951279	0.014667686	0.931220343
100130742	ENSG00000214954	100130742	LRRC69	2.217969184	-0.996343677	5.94978144	0.014719111	0.931220343
10457	ENSG00000136235	10457	GPNMB	2.986128951	5.277428932	5.928217239	0.014900309	0.931220343
6404	ENSG00000110876	6404	SELPLG	-3.247282294	2.536865597	5.927514491	0.014906252	0.931220343
8600	ENSG00000120659	8600	TNFSF11	-4.895705357	-2.771939751	5.926236223	0.014917069	0.931220343
765	ENSG00000131686	765	CA6	-6.21904638	-1.86905898	5.919086062	0.014977726	0.931220343
9429	ENSG00000118777	9429	ABCG2	-4.562755483	-0.663882865	5.912924263	0.015030202	0.931220343
25825	ENSG00000182240	25825	BACE2	-2.135395586	7.816418733	5.909024511	0.015063511	0.931220343
6695	ENSG00000152377	6695	SPOCK1	-4.639042938	2.20120504	5.904984447	0.015098099	0.931220343
9073	ENSG00000156284	9073	CLDN8	3.827041553	-1.377837968	5.904900994	0.015098814	0.931220343
100507053	ENSG00000246090	100507053	LOC100507053	2.80135279	-1.499455433	5.900553843	0.015136125	0.931220343
7851	ENSG00000144063	7851	MALL	-4.052935941	4.738804571	5.898699354	0.01515207	0.931220343
8829	ENSG00000099250	8829	NRP1	2.008992078	5.884322235	5.894471473	0.015188487	0.931220343
9770	ENSG00000101265	9770	RASSF2	-2.570599286	3.15427127	5.892561741	0.015204966	0.931220343
8685	ENSG00000019169	8685	MARCO	3.595147671	0.780283729	5.889607365	0.015230496	0.931220343
163175	ENSG00000153902	163175	LGI4	-5.44616443	-2.434675211	5.889313584	0.015233037	0.931220343
2162	ENSG00000124491	2162	F13A1	-5.238904364	0.859855944	5.885439176	0.01526659	0.931220343
81466	ENSG00000197454	81466	OR2L5	4.661117734	-2.926155643	5.869996246	0.015401085	0.931220343
619383	ENSG00000254911	619383	SCARNA9	3.522982684	-0.573043229	5.868745384	0.015412032	0.931220343
57587	ENSG00000164323	57587	CFAP97	1.21655913	5.232721405	5.865660812	0.015439062	0.931220343
116966	ENSG00000150627	116966	WDR17	2.993441382	1.351111522	5.865369957	0.015441613	0.931220343
7475	ENSG00000115596	7475	WNT6	-5.58150567	1.768875834	5.858084912	0.015505656	0.931220343
775	ENSG00000151067	775	CACNA1C	-4.128491718	1.296295845	5.854974527	0.015533083	0.931220343
9543	ENSG00000174498	9543	IGDCC3	-5.483130784	-0.654024489	5.854803183	0.015534595	0.931220343
51520	ENSG00000133706	51520	LARS1	1.214686953	7.567278985	5.846167453	0.015611013	0.931220343
6285	ENSG00000160307	6285	S100B	-5.744984441	-2.220277442	5.833942533	0.015719855	0.931220343
83887	ENSG00000120440	83887	TTLL2	-5.257120819	-2.562401014	5.826915286	0.015782775	0.931220343
7111	ENSG00000136842	7111	TMOD1	-1.583275162	7.711640538	5.821382897	0.015832492	0.931220343
3357	ENSG00000135914	3357	HTR2B	-7.412822409	-0.823880769	5.815730982	0.01588345	0.931220343
64116	ENSG00000138821	64116	SLC39A8	1.749412131	7.048316142	5.815586508	0.015884755	0.931220343
79802	ENSG00000143512	79802	HHIPL2	-7.358833819	1.90142866	5.815048935	0.015889611	0.931220343
10044	ENSG00000095370	10044	SH2D3C	-3.353177021	0.846473446	5.812376995	0.01591377	0.931220343
94026	ENSG00000158553	94026	POM121L2	3.094421986	-2.298856917	5.803443482	0.01599482	0.931220343
10223	ENSG00000143167	10223	GPA33	-4.796258726	-2.830059832	5.802644555	0.016002089	0.931220343
5068	ENSG00000172016	5068	REG3A	-7.240919645	-1.06881261	5.802552379	0.016002928	0.931220343
57408	ENSG00000144771	57408	LRTM1	-5.034034246	-2.684920586	5.795696945	0.016065445	0.931220343
11277	ENSG00000213689	11277	TREX1	-1.897027828	4.13173139	5.788165485	0.016134418	0.931220343
10804	ENSG00000121742	10804	GJB6	2.780407982	0.965626002	5.781537576	0.016195368	0.931220343
9332	ENSG00000177575	9332	CD163	2.83016273	1.726821232	5.774874545	0.016256881	0.931220343
3706	ENSG00000137825	3706	ITPKA	-3.429806399	1.249076268	5.768136165	0.016319334	0.931220343
2792	ENSG00000127928	2792	GNGT1	-5.478130395	-2.393934361	5.767922848	0.016321315	0.931220343
84676	ENSG00000158022	84676	TRIM63	-5.432800251	-2.44344558	5.759497046	0.016399766	0.931220343
3581	ENSG00000124334	3581	IL9R	4.111104251	-3.638715771	5.73514225	0.016628721	0.931220343
139599	ENSG00000186675	139599	MAGEE2	4.111104251	-3.638715771	5.73514225	0.016628721	0.931220343
728734	ENSG00000255524	728734	NPIPB8	4.111104251	-3.638715771	5.73514225	0.016628721	0.931220343
1755	ENSG00000187908	1755	DMBT1	-5.247460932	7.368241348	5.731584294	0.016662444	0.931220343
8322	ENSG00000174804	8322	FZD4	2.04816325	3.347581205	5.729459715	0.016682614	0.931220343
389421	ENSG00000187772	389421	LIN28B	-8.593491891	0.263440259	5.729268668	0.016684429	0.931220343
3273	ENSG00000113905	3273	HRG	-3.863060927	-0.900166125	5.726692713	0.016708922	0.931220343
54894	ENSG00000108375	54894	RNF43	-2.226916856	5.18215076	5.726009252	0.016715427	0.931220343

	Genes	Entrez	Symbol	logFC	logCPM	LR	PValue	FDR
389015	ENSG00000180251	389015	SLC9A4	-2.38066481	3.827202749	5.723794168	0.016736526	0.931220343
125206	ENSG00000154025	125206	SLC5A10	-5.173584036	-2.61821527	5.711647465	0.016852716	0.931220343
22885	ENSG00000173210	22885	ABLIM3	-4.320827194	4.821791683	5.698600549	0.016978445	0.931220343
26999	ENSG00000055163	26999	CYFIP2	1.402667699	6.143203271	5.692104964	0.017041401	0.931220343
6406	ENSG00000124233	6406	SEMG1	-5.781673915	-2.183071006	5.688755729	0.017073957	0.931220343
151	ENSG00000274286	151	ADRA2B	-3.973303668	1.564492175	5.685408795	0.017106554	0.931220343
84913	ENSG00000168874	84913	ATOH8	-3.574449326	1.853793316	5.684987085	0.017110665	0.931220343
185	ENSG00000144891	185	AGTR1	-8.129103206	-0.172837572	5.680012657	0.017159244	0.931220343
1360	ENSG00000153002	1360	CPB1	-7.504991622	-0.746429759	5.677097365	0.01718778	0.931220343
84226	ENSG00000221843	84226	C2orf16	-4.817466748	-2.825261757	5.673861975	0.017219506	0.931220343
9687	ENSG00000196208	9687	GREB1	1.9021035	3.448952688	5.670424434	0.017253281	0.931220343
26579	ENSG00000172927	26579	MYEOV	-5.447358857	4.280989381	5.661933035	0.017337005	0.931220343
55359	ENSG00000060140	55359	STYK1	-2.139973441	3.0617908	5.657538723	0.017380497	0.931220343
114884	ENSG00000144645	114884	OSBPL10	1.362467279	6.700315412	5.655854531	0.017397196	0.931220343
2998	ENSG00000111713	2998	GYS2	3.659562671	-3.341931158	5.653685609	0.017418725	0.931220343
1292	ENSG00000142173	1292	COL6A2	-2.19290057	7.134297411	5.643301389	0.017522183	0.931220343
9120	ENSG00000108932	9120	SLC16A6	-3.76083867	1.099738794	5.64239585	0.017531235	0.931220343
11141	ENSG00000169306	11141	IL1RAPL1	-7.512619502	-0.742995487	5.641995676	0.017535236	0.931220343
3759	ENSG00000123700	3759	KCNJ2	2.34007592	2.262948359	5.629384672	0.01766183	0.931220343
389840	ENSG00000180815	389840	MAP3K15	3.725092291	1.052459247	5.628911426	0.017666599	0.931220343
56849	ENSG00000182916	56849	TCEAL7	-5.02536831	-2.683840697	5.625603537	0.01769997	0.931220343
56300	ENSG00000136688	56300	IL36G	3.268348576	-3.188268559	5.608140259	0.017877227	0.931220343
5167	ENSG00000197594	5167	ENPP1	-3.575626222	2.634098015	5.608007089	0.017878585	0.931220343
3236	ENSG00000128710	3236	HOXD10	-5.709091912	-2.227255191	5.607224045	0.017886577	0.931220343
2731	ENSG00000178445	2731	GLDC	2.163515295	4.751218361	5.604202627	0.017917447	0.931220343
6519	ENSG00000138079	6519	SLC3A1	3.387198067	-0.019348909	5.603462801	0.017925014	0.931220343
163782	ENSG00000132854	163782	KANK4	-5.344537411	2.97893559	5.602234537	0.017937585	0.931220343
440603	ENSG00000188761	440603	BCL2L15	-3.825959134	3.477903552	5.5978223	0.017982817	0.931220343
374946	ENSG00000162490	374946	DRAXIN	-3.971510237	-1.860962614	5.596380014	0.017997628	0.931220343
8284	ENSG00000012817	8284	KDM5D	-4.925385069	-2.755972648	5.587615517	0.018087903	0.931220343
56000	ENSG00000147206	56000	NXF3	-5.505423457	0.961658334	5.586235455	0.01810216	0.931220343
57863	ENSG00000162706	57863	CADM3	4.13775883	2.535863907	5.584229968	0.018122899	0.931220343
6424	ENSG00000106483	6424	SFRP4	-4.891187569	0.096455965	5.581350478	0.01815272	0.931220343
117157	ENSG00000198574	117157	SH2D1B	-7.424692065	-0.83872221	5.576603286	0.018201993	0.931220343
27115	ENSG00000171408	27115	PDE7B	2.09776577	1.668170033	5.569818445	0.018272655	0.931220343
5992	ENSG00000111783	5992	RFX4	-9.21811691	0.861505107	5.569052016	0.018280655	0.931220343
147664	ENSG00000269526	147664	ERVV-1	5.225512721	-2.200917424	5.56886772	0.01828258	0.931220343
101929947	ENSG00000232788	101929947	ITGA6-AS1	-4.799348478	-2.83199678	5.565748024	0.018315184	0.931220343
2864	ENSG00000126266	2864	FFAR1	3.819399719	-3.702046253	5.561936784	0.018355097	0.931220343
142685	ENSG00000146809	142685	ASB15	3.819399719	-3.702046253	5.561936784	0.018355097	0.931220343
2220	ENSG00000160339	2220	FCN2	3.819399719	-3.702046253	5.561936784	0.018355097	0.931220343
127059	ENSG00000162727	127059	OR2M5	3.819399719	-3.702046253	5.561936784	0.018355097	0.931220343
343173	ENSG00000196539	343173	OR2T3	3.819399719	-3.702046253	5.561936784	0.018355097	0.931220343
613211	ENSG00000205882	613211	DEFB134	3.819399719	-3.702046253	5.561936784	0.018355097	0.931220343
101927825	ENSG00000282556	101927825	LOC101927825	3.819399719	-3.702046253	5.561936784	0.018355097	0.931220343
85376	ENSG00000275793	85376	RIMBP3	-2.997897806	-1.216571252	5.559712849	0.018378429	0.931220343
3512	ENSG00000132465	3512	JCHAIN	-5.560461083	-2.318600868	5.558819204	0.018387812	0.931220343
9848	ENSG00000198948	9848	MFAP3L	1.844774422	2.893943564	5.552524965	0.018454047	0.931220343
55762	ENSG00000167562	55762	ZNF701	1.278380559	4.914577748	5.552071192	0.018458832	0.931220343
122618	ENSG00000166428	122618	PLD4	-5.945364618	-2.068445902	5.543150076	0.018553157	0.933974678
1943	ENSG00000099617	1943	EFNA2	2.627480817	-1.215760775	5.533193938	0.018659014	0.937296511
3398	ENSG00000115738	3398	ID2	-1.637669663	4.720806089	5.521610185	0.018782962	0.940983717
623	ENSG00000100739	623	BDKRB1	-4.202100444	1.488944374	5.518864178	0.01881247	0.940983717
2571	ENSG00000128683	2571	GAD1	-4.487557342	2.26000254	5.513261142	0.018872826	0.941695405
57493	ENSG00000173706	57493	HEG1	-2.235732185	6.271132507	5.510114281	0.018906811	0.941695405
51316	ENSG00000145287	51316	PLAC8	-3.943939411	0.973316391	5.505882628	0.018952613	0.941980912
79949	ENSG00000148735	79949	PLEKHS1	-3.172996405	4.600333613	5.495361721	0.019066983	0.94423007
55214	ENSG00000090530	55214	P3H2	3.209863882	5.409366914	5.494333849	0.019078195	0.94423007
9311	ENSG00000213199	9311	ASIC3	-3.89232175	0.354021957	5.489533733	0.019130644	0.944836801
80183	ENSG00000102445	80183	RUBCNL	3.151680313	2.928744321	5.482794627	0.019204532	0.946497581

	Genes	Entrez	Symbol	logFC	logCPM	LR	PValue	FDR
353091	ENSG00000203722	353091	RAET1G	-3.01373612	-0.952047704	5.473740707	0.019304264	0.949422486
2646	ENSG00000084734	2646	GCKR	-6.387435459	-1.797225039	5.463852601	0.019413797	0.952056377
51450	ENSG00000167157	51450	PRRX2	-2.39576835	3.353149351	5.459849676	0.019458321	0.952056377
399669	ENSG00000213801	399669	ZNF321P	1.503247989	3.514750426	5.449931266	0.019569097	0.952056377
2983	ENSG00000061918	2983	GUCY1B1	2.841312452	4.692561009	5.447400122	0.019597471	0.952056377
56603	ENSG00000003137	56603	CYP26B1	-4.791936491	1.767192152	5.445299085	0.019621055	0.952056377
729475	ENSG00000214842	729475	RAD51AP2	-5.682079125	-2.252986687	5.442837013	0.01964873	0.952056377
57096	ENSG00000092200	57096	RPGRIP1	-6.885390538	-1.289509342	5.439983731	0.019680853	0.952056377
7546	ENSG00000043355	7546	ZIC2	-7.679319304	3.639433202	5.437974421	0.019703506	0.952056377
55066	ENSG00000090857	55066	PDPR	1.196595579	4.821503853	5.4362859	0.019722564	0.952056377
7412	ENSG00000162692	7412	VCAM1	-3.67241057	4.360145934	5.432727137	0.019762793	0.952056377
7681	ENSG00000179455	7681	MKRN3	-5.982736823	-0.06851731	5.42185781	0.019886189	0.956041766
54714	ENSG00000170289	54714	CNGB3	-5.434929205	-2.42152785	5.418158223	0.019928371	0.956114433
7678	ENSG00000196418	7678	ZNF124	1.435690119	5.203431744	5.41420497	0.019973547	0.95633018
389668	ENSG00000221947	389668	XKR9	-3.175048705	2.169022834	5.40395485	0.020091175	0.959058432
6947	ENSG00000134827	6947	TCN1	-6.189073594	5.231083326	5.401939433	0.020114388	0.959058432
5317	ENSG00000081277	5317	PKP1	2.672873906	1.537208803	5.398219814	0.020157302	0.959058432
84951	ENSG00000131746	84951	TNS4	-5.311175984	5.59569617	5.395070486	0.02019371	0.959058432
10417	ENSG00000159674	10417	SPON2	-3.694061409	1.671489344	5.379531101	0.020374353	0.965052023
1128	ENSG00000168539	1128	CHRM1	-4.69026119	-2.890917869	5.377164586	0.02040201	0.965052023
229	ENSG00000136872	229	ALDOB	-6.435495315	-1.644338943	5.370948567	0.02047484	0.966552238
102724770	ENSG00000278817	102724770	LOC102724770	2.922527846	-2.969906875	5.361675624	0.020583987	0.969447778
8537	ENSG00000064787	8537	BCAS1	-3.82106487	3.078960686	5.358741153	0.020618652	0.969447778
250	ENSG00000163283	250	ALPP	3.104696187	3.593967108	5.345969406	0.020770231	0.974625482
401	ENSG00000165462	401	PHOX2A	-6.043153235	-2.009698271	5.339793982	0.020843936	0.975004278
4312	ENSG00000196611	4312	MMP1	-5.726440312	6.714336406	5.336460776	0.020883831	0.975004278
79101	ENSG00000166012	79101	TAF1D	1.402019522	5.67434579	5.334884533	0.020902725	0.975004278
7903	ENSG00000113532	7903	ST8SIA4	2.214968993	3.032766634	5.327063471	0.020996733	0.975443844
5906	ENSG00000116473	5906	RAP1A	-1.283481337	5.092193478	5.324185933	0.021031431	0.975443844
2977	ENSG00000152402	2977	GUCY1A2	2.880823383	2.529341942	5.323755559	0.021036626	0.975443844
6529	ENSG00000157103	6529	SLC6A1	-6.354918231	-1.763063615	5.314477179	0.021148939	0.977117526
84419	ENSG00000166920	84419	C15orf48	-3.937784914	4.266781952	5.31064455	0.021195513	0.977117526
728441	ENSG00000133475	728441	GGT2	3.551728224	-3.090807444	5.31048856	0.021197411	0.977117526
101928941	ENSG00000234493	101928941	RHOXF1P1	3.282566711	-0.227130877	5.305198298	0.021261879	0.977136136
1750	ENSG00000006377	1750	DLX6	-8.642184734	0.307936936	5.303002482	0.021288697	0.977136136
729264	ENSG00000205456	729264	TP53TG3D	-6.799159997	-1.363825743	5.299274165	0.021334313	0.977136136
3909	ENSG00000053747	3909	LAMA3	1.616046568	9.201203417	5.295651062	0.021378738	0.977136136
4017	ENSG00000134013	4017	LOXL2	-3.240972836	6.245314052	5.293461103	0.021405637	0.977136136
80205	ENSG00000177200	80205	CHD9	1.165534699	7.071849806	5.282993912	0.021534688	0.978503488
729359	ENSG00000167676	729359	PLIN4	2.35790095	2.111971671	5.281639656	0.021551443	0.978503488
112817	ENSG00000241935	112817	HOGA1	2.684933988	0.262562193	5.280911496	0.021560458	0.978503488
2938	ENSG00000243955	2938	GSTA1	-4.349682584	1.226319891	5.264276289	0.021767472	0.979131207
126364	ENSG00000175489	126364	LRRC25	2.428860673	-1.140196208	5.252816336	0.021911279	0.979131207
7016	ENSG00000107140	7016	TESK1	-1.288700072	5.792759121	5.250812876	0.021936521	0.979131207
10656	ENSG00000131773	10656	KHDRBS3	1.40355325	4.2952843	5.250427023	0.021941386	0.979131207
388646	ENSG00000213512	388646	GBP7	-6.053680398	-1.967988886	5.248045034	0.021971443	0.979131207
55512	ENSG00000103056	55512	SMPD3	-4.478488708	5.504319138	5.244735551	0.022013274	0.979131207
388	ENSG00000143878	388	RHOB	-1.633991358	5.776873152	5.243525772	0.022028586	0.979131207
388523	ENSG00000269067	388523	ZNF728	4.290869547	1.338213064	5.243052854	0.022034575	0.979131207
4267	ENSG00000002586	4267	CD99	-1.929228391	5.938227337	5.240160826	0.022071234	0.979131207
256006	ENSG00000145700	256006	ANKRD31	2.19124239	0.092163713	5.238497513	0.022092346	0.979131207
9719	ENSG00000197859	9719	ADAMTSL2	-6.105442746	2.392197224	5.232667669	0.02216651	0.979131207
7293	ENSG00000186827	7293	TNFRSF4	-4.33964199	-1.543411369	5.229366359	0.022208622	0.979131207
51361	ENSG00000134709	51361	HOOK1	1.655804608	5.5999821	5.227369506	0.022234134	0.979131207
375057	ENSG00000203685	375057	STUM	4.788431745	0.119155031	5.225959699	0.022252164	0.979131207
10326	ENSG00000101307	10326	SIRPB1	2.674663494	3.047239337	5.222095488	0.022301662	0.979131207
171019	ENSG00000145808	171019	ADAMTS19	-4.869115913	-0.461689857	5.22152944	0.022308922	0.979131207
9715	ENSG00000159784	9715	FAM131B	-3.729489346	0.744961355	5.220907531	0.022316901	0.979131207
6582	ENSG00000112499	6582	SLC22A2	3.377277981	-3.154514851	5.219055414	0.022340683	0.979131207
163933	ENSG00000183114	163933	FAM43B	-5.511593501	-2.390198285	5.217115176	0.022365624	0.979131207

	Genes	Entrez	Symbol	logFC	logCPM	LR	PValue	FDR
729974	ENSG00000229292	729974	RFPL4AL1	4.404849235	-3.073010526	5.206310106	0.022505049	0.983403698
9518	ENSG00000130513	9518	GDF15	-3.037544749	7.682818645	5.194956788	0.022652518	0.987567698
1586	ENSG00000148795	1586	CYP17A1	-6.236158246	1.005766777	5.191596687	0.022696355	0.987567698
6545	ENSG00000099960	6545	SLC7A4	2.126147275	0.843804692	5.189300225	0.022726365	0.987567698
5125	ENSG00000099139	5125	PCSK5	-2.160389331	5.105918482	5.182365786	0.022817236	0.989261314
10717	ENSG00000134262	10717	AP4B1	-1.557465855	5.062042291	5.172016979	0.02295355	0.989261314
969	ENSG00000110848	969	CD69	2.955087194	2.285303768	5.171447306	0.022961078	0.989261314
125963	ENSG00000170929	125963	OR1M1	-5.248991811	-2.581386145	5.165894113	0.023034598	0.989261314
27035	ENSG00000007952	27035	NOX1	-4.934550686	-0.345559972	5.163661718	0.023064221	0.989261314
729238	ENSG00000185303	729238	SFTPA2	-4.364791918	-0.464445112	5.160509909	0.023106113	0.989261314
11182	ENSG00000160326	11182	SLC2A6	-2.063379417	4.306030258	5.159758155	0.023116116	0.989261314
11189	ENSG00000159409	11189	CELF3	-4.575388869	-2.957767573	5.158229608	0.02313647	0.989261314
283	ENSG00000214274	283	ANG	-2.725964574	3.451748875	5.154737842	0.023183035	0.989261314
55769	ENSG00000167766	55769	ZNF83	1.277436003	5.275455937	5.154505212	0.023186141	0.989261314
6997	ENSG00000241186	6997	TDGF1	-7.465942701	1.361912214	5.145024709	0.023313079	0.9928753
28513	ENSG00000071991	28513	CDH19	-9.863903767	1.490011671	5.1355662	0.02344044	0.994557029
79788	ENSG00000197497	79788	ZNF665	1.997899924	2.418698443	5.133727928	0.023465277	0.994557029
79861	ENSG00000178462	79861	TUBAL3	-3.453053362	-2.290333445	5.131888112	0.023490161	0.994557029
8581	ENSG00000167656	8581	LY6D	-5.987337461	1.86091673	5.129552767	0.023521788	0.994557029
83998	ENSG00000134193	83998	REG4	-6.284456246	0.959382113	5.122921116	0.023611838	0.995152112
79971	ENSG00000116729	79971	WLS	-2.842523383	8.128084952	5.119397767	0.023659826	0.995152112
6496	ENSG00000138083	6496	SIX3	-7.625709299	-0.636416196	5.119175731	0.023662854	0.995152112
275	ENSG00000145020	275	AMT	2.502657024	2.750471626	5.111662196	0.023765541	0.996884315
138046	ENSG00000184672	138046	RALYL	4.857037402	-2.410205909	5.108457498	0.02380948	0.996884315
11074	ENSG00000204616	11074	TRIM31	-6.363452949	3.415611753	5.106146541	0.023841217	0.996884315
6038	ENSG00000258818	6038	RNASE4	-3.301053115	3.415040089	5.103787494	0.02387366	0.996884315
79368	ENSG00000132704	79368	FCRL2	-7.349993755	-0.88721483	5.098008243	0.023953333	0.998437781
55343	ENSG00000181830	55343	SLC35C1	-1.733787696	5.170056573	5.094321482	0.024004303	0.998791441
7162	ENSG00000146242	7162	TPBG	-1.732835037	5.484757314	5.090626338	0.024055502	0.999153354
4583	ENSG00000198788	4583	MUC2	-3.27448512	3.247496523	5.08221186	0.024172514	0.999727753
4498	ENSG00000255986	4498	MT1JP	3.766644422	-2.985231083	5.081271922	0.024185621	0.999727753
79960	ENSG00000077684	79960	JADE1	1.124210827	4.881436141	5.080463029	0.024196907	0.999727753
401190	ENSG00000186479	401190	RGS7BP	2.671784125	-0.771372057	5.074564933	0.024279364	1
27283	ENSG00000137251	27283	TINAG	4.80918637	-2.460248926	5.071833413	0.024317651	1
2556	ENSG00000011677	2556	GABRA3	-8.798845852	0.458951586	5.067895102	0.024372962	1
238	ENSG00000171094	238	ALK	2.687236434	2.260515577	5.064376247	0.024422493	1
55450	ENSG00000162545	55450	CAMK2N1	-3.683069635	3.433979111	5.062538681	0.0244484	1
375791	ENSG00000197191	375791	CYSRT1	-3.637420472	0.27779538	5.059171458	0.024495946	1
9423	ENSG00000065320	9423	NTN1	1.939981881	5.105854604	5.057987784	0.024512683	1
29964	ENSG00000278224	29964	PRICKLE4	-2.90945839	-1.364029351	5.054872482	0.024556789	1
2352	ENSG00000110203	2352	FOLR3	3.080668536	0.35690238	5.051208816	0.024608764	1
7301	ENSG00000092445	7301	TYRO3	-1.374662067	4.885249944	5.040451461	0.024762036	1
359	ENSG00000167580	359	AQP2	-5.04341455	-2.661197815	5.029809201	0.024914643	1
1087	ENSG00000007306	1087	CEACAM7	-7.773416481	2.891104625	5.027160227	0.02495278	1
28970	ENSG00000182919	28970	C11orf54	1.209061321	5.187002536	5.022830858	0.02501524	1
51299	ENSG00000124785	51299	NRN1	-7.229646155	1.132119929	5.021931846	0.025028231	1
10734	ENSG00000066923	10734	STAG3	2.254298533	0.80826504	5.020967273	0.025042176	1
79094	ENSG00000128965	79094	CHAC1	-3.497371003	4.4058483	5.018789287	0.025073695	1
388125	ENSG00000205502	388125	C2CD4B	-6.156362741	2.827232685	5.017715061	0.025089256	1
22921	ENSG00000148450	22921	MSRB2	-1.441991557	3.930198041	5.016538192	0.025106315	1
9510	ENSG00000154734	9510	ADAMTS1	1.533165699	7.592688072	5.012095714	0.025170818	1
54913	ENSG00000178718	54913	RPP25	-2.191322083	2.132832125	5.005854449	0.025261731	1
105378616	ENSG00000229388	105378616	LINC01715	-5.062975317	-2.668951354	5.005221435	0.025270971	1
2248	ENSG00000186895	2248	FGF3	-7.105755125	-1.173156575	5.002485558	0.025310945	1
440021	ENSG00000205867	440021	KRTAP5-2	-4.942302319	-2.777385434	4.98555999	0.025559711	1
6568	ENSG00000124568	6568	SLC17A1	3.646224126	-3.739350594	4.981964535	0.025612882	1
728279	ENSG00000214518	728279	KRTAP2-2	3.646224126	-3.739350594	4.981964535	0.025612882	1
105370027	ENSG00000248636	105370027	LOC105370027	3.646224126	-3.739350594	4.981964535	0.025612882	1
256472	ENSG00000179292	256472	TMEM151A	-4.315155651	0.158533089	4.981061326	0.025626257	1
4012	ENSG00000113441	4012	LNPEP	1.284215838	6.036009931	4.97555392	0.02570797	1

	Genes	Entrez	Symbol	logFC	logCPM	LR	PValue	FDR
114770	ENSG00000161031	114770	PGLYRP2	-6.247917775	-1.814987167	4.97475409	0.02571986	1
58524	ENSG00000064218	58524	DMRT3	-6.238244853	-1.823766384	4.972710783	0.02575026	1
85569	ENSG00000197487	85569	GALP	3.486735549	-2.200881247	4.967975844	0.025820848	1
221416	ENSG00000181577	221416	C6orf223	-3.217089375	3.144705934	4.964994293	0.0258654	1
2948	ENSG00000168765	2948	GSTM4	-1.92444102	5.395777818	4.964526532	0.025872397	1
2862	ENSG00000102539	2862	MLNR	-4.88002181	-2.777715351	4.962492019	0.025902853	1
440738	ENSG00000197769	440738	MAP1LC3C	-10.93475516	2.54400092	4.959736682	0.025944158	1
89796	ENSG00000134369	89796	NAV1	-1.812218664	5.87506746	4.958744758	0.025959044	1
53831	ENSG00000139572	53831	GPR84	4.838933611	-2.381208785	4.956787502	0.025988444	1
57639	ENSG00000135205	57639	CCDC146	2.509031795	6.160790036	4.954707446	0.026019727	1
84873	ENSG00000144820	84873	ADGRG7	-4.289899946	-3.104334588	4.954623609	0.026020989	1
344787	ENSG00000197385	344787	ZNF860	1.866298793	4.079430972	4.953170035	0.026042874	1
118429	ENSG00000163297	118429	ANTXR2	-4.163684434	4.013138324	4.943154638	0.026194187	1
3868	ENSG00000186832	3868	KRT16	-4.189718561	5.220215511	4.938972079	0.026257648	1
101929335	ENSG00000241158	101929335	ADAMTS9-AS1	3.387908083	-1.641006581	4.938595657	0.026263367	1
11199	ENSG00000109511	11199	ANXA10	-8.766437367	4.468063665	4.928492727	0.02641735	1
7350	ENSG00000109424	7350	UCP1	-4.749762251	0.673940104	4.92843679	0.026418205	1
60437	ENSG00000124215	60437	CDH26	2.477576725	0.258586477	4.926799354	0.026443251	1
3678	ENSG00000161638	3678	ITGA5	-3.078853633	5.326527129	4.918922201	0.026564084	1
2622	ENSG00000141013	2622	GAS8	1.230352536	4.515405079	4.917447779	0.026586765	1
4653	ENSG00000034971	4653	MYOC	3.897009799	0.527208518	4.917078907	0.026592442	1
860	ENSG00000124813	860	RUNX2	-2.977406258	3.187413892	4.916867762	0.026595693	1
138715	ENSG00000205143	138715	ARID3C	2.163674217	-1.282484375	4.916822927	0.026596383	1
387104	ENSG00000255330	387104	SOGA3	-4.355562205	0.603184396	4.916718256	0.026597994	1
9427	ENSG00000171551	9427	ECEL1	-2.730715368	0.335945193	4.907512408	0.026740125	1
80328	ENSG00000131015	80328	ULBP2	-3.435173353	2.379618848	4.906636244	0.026753693	1
10141	ENSG00000082929	10141	LINC01587	-5.981629565	-2.081358135	4.892782418	0.026969189	1
2624	ENSG00000179348	2624	GATA2	-4.502562059	2.126635634	4.892050831	0.026980619	1
81623	ENSG00000125788	81623	DEFB126	-5.921393457	-2.070145093	4.889476674	0.027020875	1
26996	ENSG00000173890	26996	GPR160	2.260929064	3.173406356	4.887537842	0.027051238	1
8516	ENSG00000077943	8516	ITGA8	-5.325873213	-0.798929116	4.879587069	0.02717612	1
219855	ENSG00000134955	219855	SLC37A2	2.142331423	-0.89771815	4.877703658	0.02720579	1
283576	ENSG00000177108	283576	ZDHHC22	-4.459769467	-3.016884883	4.877034649	0.027216337	1
4680	ENSG00000086548	4680	CEACAM6	-5.268420607	8.543947377	4.876661204	0.027222227	1
84733	ENSG00000173894	84733	CBX2	-1.938634611	3.830464068	4.87497446	0.027248844	1
85416	ENSG00000139800	85416	ZIC5	-7.291188893	2.033038777	4.8742632	0.027260076	1
221188	ENSG00000159618	221188	ADGRG5	-6.158770892	-1.888061679	4.873506236	0.027272035	1
4069	ENSG00000090382	4069	LYZ	-4.181431513	4.139538788	4.872787163	0.027283401	1
412	ENSG00000101846	412	STS	-2.074346767	5.405681047	4.868225319	0.027355619	1
4061	ENSG00000160932	4061	LY6E	-2.585674179	8.182424904	4.865082557	0.027405487	1
750	ENSG00000221819	750	GAS8-AS1	3.58047113	-3.749071329	4.854960419	0.027566746	1
57414	ENSG00000005486	57414	RHBDD2	-1.307922519	6.582371631	4.851426164	0.027623284	1
671	ENSG00000101425	671	BPI	-5.381124884	-2.458003123	4.85141945	0.027623391	1
10551	ENSG00000106541	10551	AGR2	-2.379757628	8.028811102	4.848285605	0.027673625	1
100996301	ENSG00000230798	100996301	FOXD3-AS1	-4.778883025	-2.839053905	4.841002936	0.027790728	1
2516	ENSG00000136931	2516	NR5A1	-7.495946429	-0.750027608	4.836502145	0.027863357	1
79026	ENSG00000124942	79026	AHNAK	1.19708075	13.01565383	4.834277032	0.027899336	1
7075	ENSG00000066056	7075	TIE1	-5.401107865	3.173865498	4.82377932	0.028069735	1
388282	ENSG00000187185	388282	LOC388282	3.465645591	-0.472932541	4.823341458	0.028076866	1
23321	ENSG00000109654	23321	TRIM2	-1.626469599	8.479081823	4.823125664	0.028080381	1
5577	ENSG00000005249	5577	PRKAR2B	2.037364439	3.581870667	4.821797352	0.028102027	1
79838	ENSG00000103534	79838	TMC5	-4.214329593	7.756682322	4.817070385	0.028179201	1
3595	ENSG00000081985	3595	IL12RB2	3.272381478	-0.959026611	4.812145381	0.028259842	1
1048	ENSG00000105388	1048	CEACAM5	-5.189703641	7.828434008	4.811252729	0.028274484	1
148753	ENSG00000143340	148753	FAM163A	-5.746266255	-2.163610773	4.797440601	0.028502048	1
51280	ENSG00000135052	51280	GOLM1	-1.407960211	7.207292898	4.794091078	0.028557521	1
157697	ENSG00000104714	157697	ERICH1	1.203926763	4.710833903	4.792015585	0.02859195	1
256933	ENSG00000183979	256933	NPB	-4.510725944	-2.989331736	4.791982911	0.028592492	1
10720	ENSG00000213759	10720	UGT2B11	-6.407054902	-1.686375348	4.79179162	0.028595668	1
3169	ENSG00000129514	3169	FOXA1	-5.179379926	4.483389766	4.788424109	0.028651629	1

	Genes	Entrez	Symbol	logFC	logCPM	LR	PValue	FDR
57121	ENSG00000184574	57121	LPAR5	-3.006862605	0.206906537	4.780059783	0.028791121	1
23452	ENSG00000136859	23452	ANGPTL2	-2.971317661	3.405276553	4.778135692	0.028823309	1
25943	ENSG00000088854	25943	C20orf194	1.230320834	4.137846249	4.773946223	0.028893524	1
11006	ENSG00000186818	11006	LILRB4	3.046101213	-0.589610281	4.772389791	0.028919655	1
1306	ENSG00000204291	1306	COL15A1	-3.670641278	2.033264354	4.770757039	0.028947094	1
367	ENSG00000169083	367	AR	2.398251326	4.842491109	4.767677579	0.028998919	1
91522	ENSG00000050767	91522	COL23A1	2.911608245	2.29666365	4.764607052	0.02905069	1
339400	ENSG00000237975	339400	FLG-AS1	3.184596553	0.963755581	4.76234431	0.029088903	1
10265	ENSG00000176842	10265	IRX5	3.22035601	2.073081043	4.761586577	0.029101711	1
51313	ENSG00000164125	51313	GASK1B	-3.022327205	4.395756662	4.760937122	0.029112693	1
1230	ENSG00000163823	1230	CCR1	3.15391647	-0.739166014	4.758963011	0.029146103	1
23676	ENSG00000091482	23676	SMPX	-4.301740456	-1.68916154	4.758635639	0.029151647	1
10814	ENSG00000145920	10814	CPLX2	-4.704712849	-0.623460278	4.758242426	0.029158308	1
10469	ENSG00000104980	10469	TIMM44	1.283595196	5.416179094	4.756014063	0.029196085	1
9576	ENSG00000077327	9576	SPAG6	-7.169123653	-1.043102978	4.753914941	0.029231717	1
284422	ENSG00000095932	284422	SMIM24	-3.320796095	2.323905274	4.753912285	0.029231762	1
722	ENSG00000123838	722	C4BPA	-6.780552287	5.795492391	4.752537483	0.029255124	1
43849	ENSG00000186474	43849	KLK12	-5.069907231	-2.686336025	4.752062785	0.029263195	1
83468	ENSG00000120820	83468	GLT8D2	-4.168727091	1.076823275	4.747360216	0.029343275	1
10086	ENSG00000132297	10086	HHLA1	3.322997702	-3.445114907	4.747268411	0.029344841	1
3034	ENSG00000084110	3034	HAL	-3.456865938	-0.56884096	4.747213084	0.029345785	1
1357	ENSG00000091704	1357	CPA1	-6.440519304	-1.659661346	4.743706062	0.02940566	1
64388	ENSG00000180875	64388	GREM2	-5.027501344	-2.717473812	4.740318027	0.029463624	1
149708	ENSG00000175121	149708	WFDC5	3.235553187	-3.474291729	4.738190771	0.029500079	1
145258	ENSG00000133937	145258	GSC	-5.205045099	-0.943172365	4.733009022	0.029589077	1
30009	ENSG00000073861	30009	TBX21	3.513178025	-3.758815962	4.729120936	0.029656039	1
4490	ENSG00000169688	4490	MT1B	3.513178025	-3.758815962	4.729120936	0.029656039	1
92369	ENSG00000175093	92369	SPSB4	-8.336259532	0.020674317	4.728846477	0.029660771	1
5409	ENSG00000141744	5409	PNMT	-3.927620612	-0.788462662	4.724748903	0.029731524	1
65108	ENSG00000175130	65108	MARCKSL1	-1.257618742	7.258484055	4.722704103	0.029766897	1
66004	ENSG00000180155	66004	LYNX1	-2.941012957	6.056417974	4.715693769	0.029888503	1
162966	ENSG00000189190	162966	ZNF600	1.251461868	5.170788088	4.714926824	0.029901838	1
25987	ENSG00000182704	25987	TSKU	-2.005034563	5.157550771	4.712942272	0.029936374	1
4103	ENSG00000147381	4103	MAGEA4	-8.225344785	2.11249725	4.709963129	0.029988295	1
102724560	ENSG00000274276	102724560	CBSL	2.548372918	1.777584392	4.703655365	0.030098538	1
142680	ENSG00000198569	142680	SLC34A3	-3.109489265	-2.518834607	4.695410622	0.030243272	1
55714	ENSG00000218336	55714	TENM3	3.471546393	5.901850275	4.691981681	0.030303679	1
653316	ENSG00000204677	653316	FAM153CP	2.364729239	-1.961852014	4.687901947	0.030375715	1
1271	ENSG00000122756	1271	CNTFR	-4.998993367	0.866619624	4.677433207	0.03056138	1
27290	ENSG00000122711	27290	SPINK4	-6.113785809	-1.890766734	4.676800563	0.030572638	1
1303	ENSG00000111799	1303	COL12A1	2.415458259	8.5789564	4.675299602	0.030599365	1
363	ENSG00000086159	363	AQP6	-4.76116609	-1.1720678	4.668224835	0.03072567	1
2859	ENSG00000178623	2859	GPR35	-3.26601897	0.278192551	4.666075624	0.030764148	1
5341	ENSG00000115956	5341	PLEK	2.5570582	-1.367822044	4.662238914	0.030832961	1
10107	ENSG00000204613	10107	TRIM10	-4.443110881	-0.191165416	4.658215707	0.030905292	1
285016	ENSG00000189292	285016	ALKAL2	3.144092598	1.657360447	4.654733449	0.03096804	1
26095	ENSG00000204179	26095	PTPN20	3.322939526	-0.622989269	4.65288607	0.031001383	1
11170	ENSG00000168309	11170	FAM107A	1.929575698	3.158108235	4.648863277	0.031074118	1
145482	ENSG00000140043	145482	PTGR2	1.384375262	3.724136326	4.645459013	0.031135809	1
5524	ENSG00000119383	5524	PTPA	-1.280933805	7.012262647	4.643290165	0.031175179	1
9639	ENSG00000104728	9639	ARHGEF10	1.206980659	5.875802259	4.640088246	0.031233396	1
27131	ENSG00000089006	27131	SNX5	1.027431484	6.7071511	4.63557303	0.031315685	1
10219	ENSG00000139187	10219	KLRG1	-3.202701441	-1.447314028	4.631576344	0.031388712	1
9502	ENSG00000155622	9502	XAGE2	-7.030272569	-1.170583998	4.626057088	0.031489852	1
91937	ENSG00000145850	91937	TIMD4	4.459103742	-1.988964169	4.619644405	0.03160779	1
222183	ENSG00000177679	222183	SRRM3	-3.92431807	-1.900034659	4.618529436	0.031628343	1
4313	ENSG00000087245	4313	MMP2	-3.223048902	6.967868816	4.616823086	0.031659824	1
80341	ENSG00000078898	80341	BPIFB2	2.481589265	-2.335414941	4.616065265	0.031673816	1
3371	ENSG00000041982	3371	TNC	-2.608024913	8.058583643	4.615891684	0.031677022	1
23520	ENSG00000248546	23520	ANP32C	2.32986415	-1.855892365	4.61294593	0.031731477	1

	Genes	Entrez	Symbol	logFC	logCPM	LR	PValue	FDR
3814	ENSG00000170498	3814	KISS1	-2.988837765	-1.615299607	4.612043292	0.031748183	1
9670	ENSG00000117408	9670	IPO13	-1.432475552	5.149022617	4.608699187	0.031810154	1
2350	ENSG00000165457	2350	FOLR2	2.531810765	-2.276210519	4.60575015	0.031864909	1
146556	ENSG00000153446	146556	C16orf89	-4.571798573	-2.951521969	4.605061133	0.031877717	1
1178	ENSG00000105205	1178	CLC	3.444388188	-3.768584205	4.604652202	0.03188532	1
100129385	ENSG00000205549	100129385	C9orf92	3.444388188	-3.768584205	4.604652202	0.03188532	1
646282	ENSG00000214313	646282	AZGP1P1	3.444388188	-3.768584205	4.604652202	0.03188532	1
342666	ENSG00000214553	342666	LRRC37A11P	3.444388188	-3.768584205	4.604652202	0.03188532	1
100507651	ENSG00000253301	100507651	LINC01606	3.444388188	-3.768584205	4.604652202	0.03188532	1
100775107	ENSG00000281691	100775107	RBM5-AS1	3.444388188	-3.768584205	4.604652202	0.03188532	1
5540	ENSG00000204174	5540	NPY4R	-6.388145509	-1.73148425	4.604628939	0.031885753	1
9528	ENSG00000116209	9528	TMEM59	-1.456641633	8.427120598	4.598162177	0.032006249	1
5521	ENSG00000156475	5521	PPP2R2B	2.778211792	1.861419239	4.597980741	0.032009636	1
29066	ENSG00000122299	29066	ZC3H7A	1.021901495	6.15014912	4.595399217	0.032057876	1
23619	ENSG00000269699	23619	ZIM2	3.003601442	-1.06623357	4.593992212	0.0320842	1
126129	ENSG00000169169	126129	CPT1C	-4.696976305	2.98825591	4.591568746	0.032129594	1
219749	ENSG00000175395	219749	ZNF25	1.482462375	3.436825306	4.587810052	0.032200131	1
83886	ENSG00000172382	83886	PRSS27	-2.835699913	-0.834796812	4.583698346	0.032277478	1
96459	ENSG00000217128	96459	FNIP1	1.040167275	6.585991167	4.580582477	0.032336221	1
387856	ENSG00000177875	387856	CCDC184	-3.079939966	-0.749487316	4.579792129	0.032351139	1
349136	ENSG00000187260	349136	WDR86	-3.460478727	1.592606039	4.579526336	0.032356158	1
56108	ENSG00000253537	56108	PCDHGA7	-2.658799449	1.829599478	4.578233594	0.032380578	1
3604	ENSG00000049249	3604	TNFRSF9	-4.386007136	2.618094397	4.576182862	0.032419356	1
5226	ENSG00000142657	5226	PGD	-1.534986943	7.712072543	4.57530153	0.032436037	1
80045	ENSG00000180758	80045	GPR157	-2.381671454	2.058594699	4.573911303	0.032462367	1
7080	ENSG00000136352	7080	NKX2-1	-4.312481014	-3.090099229	4.572236292	0.032494121	1
339896	ENSG00000144644	339896	GADL1	4.086799112	-2.584572169	4.572215929	0.032494507	1
93649	ENSG00000141052	93649	MYOCD	2.78166539	-2.71434459	4.570342726	0.032530057	1
113278	ENSG00000101276	113278	SLC52A3	-2.271110787	4.646266602	4.568870096	0.032558033	1
5159	ENSG00000113721	5159	PDGFRB	-3.707645232	2.054917639	4.567522637	0.032583653	1
10216	ENSG00000116690	10216	PRG4	-5.97955268	1.916712441	4.564916627	0.032633263	1
441282	ENSG00000227471	441282	AKR1B15	-7.508426841	2.147082074	4.564837784	0.032634765	1
4005	ENSG00000135363	4005	LMO2	-2.978918812	1.467901699	4.562585559	0.032677705	1
84986	ENSG00000213390	84986	ARHGAP19	1.295914765	4.201461825	4.562195114	0.032685155	1
118427	ENSG00000118733	118427	OLFM3	-5.09642305	-2.637477266	4.560072922	0.03272568	1
1282	ENSG00000187498	1282	COL4A1	1.910601448	8.635159533	4.558649368	0.032752893	1
256158	ENSG00000148357	256158	HMCN2	-5.335668703	0.636741655	4.556457346	0.032794843	1
10810	ENSG00000132970	10810	WASF3	2.141679581	3.886864117	4.55458856	0.032830651	1
6506	ENSG00000110436	6506	SLC1A2	-3.060064552	-1.518485874	4.551487553	0.03289016	1
1761	ENSG00000137090	1761	DMRT1	-4.807679552	-1.13242939	4.549245746	0.032933251	1
9992	ENSG00000159197	9992	KCNE2	2.932009797	-2.525210037	4.547840681	0.032960288	1
2297	ENSG00000251493	2297	FOXD1	-4.602915799	-0.698182146	4.546949399	0.032977451	1
4239	ENSG00000166482	4239	MFAP4	-5.082153188	1.931473525	4.544839383	0.03301812	1
140469	ENSG00000071909	140469	MYO3B	1.522132439	4.287679679	4.544727879	0.03302027	1
100132247	ENSG00000243716	100132247	NPIPB5	1.311869016	5.221418041	4.540063577	0.033110362	1
79230	ENSG00000130544	79230	ZNF557	1.17972165	3.95027442	4.53992332	0.033113075	1
8277	ENSG00000007350	8277	TKTL1	-8.44472098	0.122927438	4.539837372	0.033114738	1
816	ENSG00000058404	816	CAMK2B	2.341865149	-0.218553546	4.53922885	0.033126512	1
152756	ENSG00000250486	152756	FAM218A	2.196379229	-0.336529057	4.534066973	0.033226565	1
85439	ENSG00000140022	85439	STON2	1.847429628	6.361597386	4.53395383	0.033228762	1
2826	ENSG00000184451	2826	CCR10	-3.251915195	-1.831644005	4.531605307	0.033274391	1
8294	ENSG00000276180	8294	H4C9	1.709229799	4.727554133	4.530612407	0.033293701	1
5618	ENSG00000113494	5618	PRLR	2.567258433	0.014391638	4.530330281	0.033299191	1
56547	ENSG00000167346	56547	MMP26	-9.972080663	1.57125049	4.527755759	0.033349325	1
7503	ENSG00000229807	7503	XIST	-6.029866079	-2.013427505	4.527386142	0.033356529	1
89885	ENSG00000147378	89885	FATE1	-4.615939448	-2.939853493	4.519989959	0.033501028	1
90333	ENSG00000204604	90333	ZNF468	1.201938329	5.945252403	4.502393497	0.033847445	1
3082	ENSG00000019991	3082	HGF	-4.081034711	0.005667881	4.501757379	0.033860038	1
896	ENSG00000112576	896	CCND3	-1.811640125	5.178794853	4.500117241	0.03389253	1
22915	ENSG00000138722	22915	MMRN1	-5.249166052	-2.540379822	4.499287933	0.033908971	1

	Genes	Entrez	Symbol	logFC	logCPM	LR	PValue	FDR
25805	ENSG00000095739	25805	BAMBI	-3.910574965	4.021592058	4.496306825	0.033968141	1
4747	ENSG00000277586	4747	NEFL	-7.123725529	1.869947827	4.495875213	0.033976717	1
165530	ENSG00000152672	165530	CLEC4F	-4.18861723	-3.149905321	4.49581995	0.033977815	1
26257	ENSG00000136327	26257	NKX2-8	-4.506131066	-2.987141032	4.494983789	0.033994436	1
8728	ENSG00000135074	8728	ADAM19	-3.151624112	5.52688572	4.488712208	0.034119371	1
170958	ENSG00000203326	170958	ZNF525	1.467261217	4.496901949	4.488483003	0.034123946	1
127687	ENSG00000197982	127687	C1orf122	-1.671708618	3.399353036	4.488148916	0.034130615	1
404037	ENSG00000187664	404037	HAPLN4	-5.110100682	-2.635878062	4.482922641	0.034235129	1
84970	ENSG00000142698	84970	C1orf94	-5.797811972	-2.158732537	4.47701483	0.034353675	1
84885	ENSG00000160446	84885	ZDHHC12	-1.402443502	4.614867383	4.476729522	0.034359411	1
201294	ENSG00000092929	201294	UNC13D	-3.716827419	5.93391089	4.476415624	0.034365722	1
100506736	ENSG00000205045	100506736	SLFN12L	-3.186778309	-1.359255601	4.467127688	0.034553031	1
339768	ENSG00000144488	339768	ESPNL	2.675453256	-0.035737604	4.455576321	0.034787476	1
84239	ENSG00000127249	84239	ATP13A4	-4.182655478	1.745743613	4.454928117	0.034800681	1
3101	ENSG00000160883	3101	HK3	2.233540177	-1.883675906	4.453509486	0.034829599	1
283869	ENSG00000183971	283869	NPW	-3.786768978	-1.340555695	4.452210471	0.034856101	1
2015	ENSG00000174837	2015	ADGRE1	2.686344353	-0.356363549	4.447625616	0.034949808	1
100874014	ENSG00000254453	100874014	NAV2-AS2	-3.909600787	-3.27168093	4.445817325	0.034986839	1
27347	ENSG00000198648	27347	STK39	1.999564606	6.807185036	4.444743774	0.035008843	1
80820	ENSG00000122547	80820	EEPD1	-1.742467579	3.749736428	4.43964635	0.03511352	1
23464	ENSG00000100116	23464	GCAT	1.30285287	3.363465524	4.433317215	0.035243947	1
10317	ENSG00000183778	10317	B3GALT5	-4.297056599	2.144014455	4.431052258	0.035290744	1
5784	ENSG00000152104	5784	PTPN14	1.250073732	8.031108411	4.421854349	0.035481457	1
50945	ENSG00000122145	50945	TBX22	3.032605279	-3.04446841	4.415603647	0.035611677	1
7368	ENSG00000174607	7368	UGT8	-2.394890389	4.865940891	4.413125782	0.035663436	1
91156	ENSG00000163395	91156	IGFN1	-7.337825039	7.358752801	4.412984621	0.035666387	1
89870	ENSG00000204610	89870	TRIM15	-3.662595583	2.313290801	4.404642413	0.035841238	1
4018	ENSG00000198670	4018	LPA	3.531027567	-1.870897671	4.403548661	0.035864229	1
8913	ENSG00000006283	8913	CACNA1G	-3.776843783	-0.194654754	4.400468129	0.035929067	1
8242	ENSG00000126012	8242	KDM5C	-1.186516802	7.888771762	4.397607661	0.035989382	1
342931	ENSG00000223638	342931	RFPL4A	4.58147533	-1.341058941	4.397192103	0.035998154	1
846	ENSG00000036828	846	CASR	3.873247407	-2.696224563	4.388747379	0.036176883	1
6440	ENSG00000168484	6440	SFTPC	-3.579976818	-2.149969828	4.388170154	0.036189134	1
285386	ENSG00000188001	285386	TPRG1	1.944356888	-0.348719927	4.387008302	0.036213805	1
29988	ENSG00000136856	29988	SLC2A8	-1.629075466	2.885129212	4.386714486	0.036220047	1
10110	ENSG00000101049	10110	SGK2	1.979896861	1.111332681	4.367523477	0.036630192	1
10859	ENSG00000104972	10859	LILRB1	2.627658193	-0.822213158	4.364646179	0.036692102	1
5798	ENSG00000054356	5798	PTPRN	-4.025377291	-0.131610503	4.358109546	0.036833158	1
2012	ENSG00000134531	2012	EMP1	-1.646130316	5.430268736	4.348871181	0.037033485	1
8544	ENSG00000087842	8544	PIR	-1.768645609	2.826840008	4.347721437	0.037058496	1
376267	ENSG00000139998	376267	RAB15	-2.085745329	2.662432058	4.344585498	0.037126804	1
54102	ENSG00000159212	54102	CLIC6	-3.465502461	2.980475234	4.342330218	0.03717601	1
64282	ENSG00000121274	64282	TENT4B	1.243907738	5.890910841	4.339214203	0.037244109	1
56479	ENSG00000185760	56479	KCNQ5	3.738722731	0.501280062	4.332716892	0.037386525	1
118611	ENSG00000154493	118611	C10orf90	-4.608368883	-2.937864681	4.331400667	0.037415445	1
6364	ENSG00000115009	6364	CCL20	-3.053997999	7.344612879	4.330556147	0.037434013	1
1301	ENSG00000060718	1301	COL11A1	-6.033652476	3.10748809	4.328373833	0.037482039	1
63976	ENSG00000142611	63976	PRDM16	-3.852705139	2.000815425	4.326259955	0.037528621	1
146713	ENSG00000167281	146713	RBFOX3	-4.561474428	0.897697129	4.322477254	0.037612128	1
55966	ENSG00000196581	55966	AJAP1	2.348537621	4.331462372	4.316716942	0.037739668	1
171389	ENSG00000174885	171389	NLRP6	-4.386018905	-3.066258196	4.312369097	0.037836235	1
7545	ENSG00000152977	7545	ZIC1	-7.960550192	4.388876815	4.31123245	0.037861522	1
28966	ENSG00000064652	28966	SNX24	1.328248872	4.340795602	4.311070033	0.037865137	1
56288	ENSG00000148498	56288	PARD3	1.061443856	7.56012723	4.309747922	0.037894576	1
5654	ENSG00000166033	5654	HTRA1	1.902734176	4.129585451	4.307383345	0.037947288	1
2627	ENSG00000141448	2627	GATA6	-3.217954314	5.302267573	4.306316775	0.037971089	1
6783	ENSG00000109193	6783	SULT1E1	-7.187948113	-1.035701572	4.302506119	0.038056254	1
105369154	ENSG00000270580	105369154	PKD1P6-NPIPP1	2.330011309	0.144696146	4.302241475	0.038062176	1
30850	ENSG00000109089	30850	CDR2L	-1.289249967	4.728152111	4.301378167	0.038081501	1
80255	ENSG00000115084	80255	SLC35F5	0.972300246	6.534629032	4.297383383	0.038171059	1

	Genes	Entrez	Symbol	logFC	logCPM	LR	PValue	FDR
9235	ENSG00000008517	9235	IL32	-2.745628134	5.414177389	4.289402091	0.03835065	1
2173	ENSG00000164434	2173	FABP7	-9.675023871	1.305368805	4.289287509	0.038353235	1
389792	ENSG00000188483	389792	IER5L	-2.850589361	5.557371709	4.287600764	0.038391305	1
63895	ENSG00000154864	63895	PIEZO2	-5.605921636	4.174617359	4.287507633	0.038393408	1
2053	ENSG00000120915	2053	EPHX2	1.704081961	3.951953676	4.287083538	0.038402987	1
6263	ENSG00000198838	6263	RYR3	2.985171117	1.253450043	4.282024904	0.038517436	1
51738	ENSG00000157017	51738	GHRL	2.431339993	-2.606150418	4.280989562	0.038540904	1
477	ENSG00000018625	477	ATP1A2	-4.689333325	3.745933474	4.279927398	0.038564996	1
1831	ENSG00000157514	1831	TSC22D3	-3.060872864	6.161362229	4.277268226	0.038625379	1
124842	ENSG00000181291	124842	TMEM132E	-7.67282227	-0.623410557	4.277091656	0.038629392	1
286343	ENSG00000153714	286343	LURAP1L	-2.459808803	2.2359975	4.27652136	0.038642357	1
2636	ENSG00000164900	2636	GBX1	-4.600310383	-2.937705756	4.274361158	0.038691506	1
8399	ENSG00000069764	8399	PLA2G10	-3.789339472	-0.892903545	4.272749763	0.038728211	1
63827	ENSG00000132692	63827	BCAN	-4.957004406	1.419965168	4.270911473	0.038770129	1
9550	ENSG00000136888	9550	ATP6V1G1	-1.020864658	6.615169368	4.269448392	0.038803525	1
7620	ENSG00000198429	7620	ZNF69	2.069629807	3.7384917	4.268013038	0.038836318	1
22824	ENSG00000164070	22824	HSPA4L	1.436200124	4.206650912	4.262893227	0.038953524	1
5034	ENSG00000185624	5034	P4HB	-1.192191412	9.148343844	4.261625329	0.038982607	1
148418	ENSG00000203943	148418	SAMD13	-2.443176838	-0.912690866	4.260276933	0.039013561	1
221830	ENSG00000105849	221830	TWISTNB	1.307041959	4.149204517	4.259122644	0.03904008	1
3284	ENSG00000203859	3284	HSD3B2	-8.327450055	0.034322989	4.255856472	0.039115219	1
7433	ENSG00000114812	7433	VIPR1	-3.424744376	3.162758092	4.254775612	0.039140119	1
5671	ENSG00000221826	5671	PSG3	-4.60660092	-2.934555702	4.251186877	0.039222909	1
479	ENSG00000075673	479	ATP12A	2.687304425	-0.543421419	4.250875817	0.039230094	1
10344	ENSG00000006606	10344	CCL26	-4.586108086	-2.94695635	4.248910452	0.039275521	1
6511	ENSG00000105143	6511	SLC1A6	-4.875397794	-2.782644444	4.248017527	0.039296178	1
583	ENSG00000125124	583	BBS2	1.008617234	6.000325714	4.24772127	0.039303034	1
190	ENSG00000169297	190	NR0B1	-6.075931238	0.028562072	4.244973548	0.039366683	1
55184	ENSG00000089091	55184	DZANK1	1.383503527	2.85037276	4.243729063	0.039395546	1
79962	ENSG00000178401	79962	DNAJC22	-3.227073505	3.604670675	4.241404305	0.039449524	1
56901	ENSG00000185633	56901	NDUFA4L2	-3.869926678	2.121080208	4.240441494	0.039471901	1
6351	ENSG00000275302	6351	CCL4	3.05257519	-0.574369734	4.234369988	0.039613323	1
81553	ENSG00000197872	81553	FAM49A	1.777379758	1.697439726	4.232599359	0.039654666	1
2525	ENSG00000171124	2525	FUT3	-4.116574963	5.245899127	4.232348422	0.039660529	1
79848	ENSG00000104218	79848	CSPP1	1.341824206	4.706180521	4.231590495	0.039678242	1
7031	ENSG00000160182	7031	TFF1	-5.979617025	4.523624356	4.229584718	0.039725159	1
339500	ENSG00000181450	339500	ZNF678	1.090690011	4.723354832	4.228884099	0.039741561	1
10855	ENSG00000173083	10855	HPSE	-2.429783075	3.564880935	4.228301207	0.039755212	1
2259	ENSG00000102466	2259	FGF14	3.297199033	-1.793837933	4.226822949	0.039789855	1
3646	ENSG00000104408	3646	EIF3E	1.0083271	8.808942137	4.225763341	0.039814706	1
653808	ENSG00000174992	653808	ZG16	-5.779187946	-2.152007149	4.223572752	0.039866134	1
150165	ENSG00000172967	150165	XKR3	3.763410083	-3.30101198	4.216099766	0.040042101	1
10000	ENSG00000117020	10000	AKT3	2.74248242	3.811796868	4.213950414	0.040092863	1
160419	ENSG00000165805	160419	C12orf50	3.119902845	-3.500047248	4.211256566	0.04015658	1
80352	ENSG00000204618	80352	RNF39	-3.733961478	3.704013061	4.202046431	0.040375228	1
284611	ENSG00000162636	284611	FAM102B	-1.992052413	3.150536829	4.19983961	0.040427803	1
115701	ENSG00000198796	115701	ALPK2	-3.55545897	2.331125916	4.191964055	0.040616016	1
11024	ENSG00000104974	11024	LILRA1	3.015845104	-2.649677397	4.191901895	0.040617505	1
3316	ENSG00000170276	3316	HSPB2	2.237094367	1.058262271	4.188583014	0.0406971	1
79742	ENSG00000147113	79742	DIPK2B	-4.607560483	-2.946834845	4.184522153	0.040794712	1
1003	ENSG00000179776	1003	CDH5	-3.895905208	4.303876237	4.183572899	0.040817564	1
344905	ENSG00000187527	344905	ATP13A5	-4.684711485	-2.899296486	4.180212021	0.040898584	1
56165	ENSG00000095627	56165	TDRD1	-3.788677964	-1.263877892	4.17821544	0.040946795	1
1144	ENSG00000135902	1144	CHRND	-4.576330204	-2.978473423	4.175668274	0.041008387	1
257019	ENSG00000172159	257019	FRMD3	-2.151470875	2.374045442	4.172840833	0.041076871	1
5646	ENSG00000010438	5646	PRSS3	-3.76706588	0.646815341	4.167831696	0.041198492	1
8744	ENSG00000125657	8744	TNFSF9	-4.893746845	5.392734556	4.165822478	0.041247382	1
11149	ENSG00000112276	11149	BVES	-2.774583941	1.649158871	4.163610033	0.041301288	1
5979	ENSG00000165731	5979	RET	2.232052859	1.953136705	4.163397149	0.041306479	1
136259	ENSG00000266265	136259	KLF14	-4.572706347	-2.951587385	4.163345981	0.041307726	1

	Genes	Entrez	Symbol	logFC	logCPM	LR	PValue	FDR
9771	ENSG00000136237	9771	RAPGEF5	1.664859046	5.407099172	4.159623066	0.041398616	1
1311	ENSG00000105664	1311	COMP	-3.03686487	0.369079571	4.158304434	0.041430859	1
5266	ENSG00000124102	5266	PI3	1.794251756	6.19360826	4.155462608	0.041500436	1
8912	ENSG00000196557	8912	CACNA1H	2.274633309	1.528399336	4.155287019	0.041504739	1
1588	ENSG00000137869	1588	CYP19A1	-4.780935123	-2.811910667	4.155053	0.041510475	1
2196	ENSG00000086570	2196	FAT2	1.731024085	6.894805784	4.154665467	0.041519975	1
79852	ENSG00000105131	79852	EPHX3	-3.420455129	0.548128272	4.153995409	0.041536406	1
4317	ENSG00000118113	4317	MMP8	3.846417899	-1.851570075	4.148802001	0.041663991	1
9507	ENSG00000158859	9507	ADAMTS4	-3.068711513	0.074392351	4.145916289	0.041735062	1
23167	ENSG00000132294	23167	EFR3A	1.211029816	7.169586112	4.141295887	0.041849121	1
7415	ENSG00000165280	7415	VCP	-1.132883832	9.299033579	4.140777074	0.041861948	1
55786	ENSG00000170954	55786	ZNF415	1.50878978	3.535295365	4.13614501	0.041976661	1
4620	ENSG00000125414	4620	MYH2	2.650436674	-1.581447578	4.135237073	0.041999184	1
56262	ENSG00000136802	56262	LRRC8A	-1.666727296	6.979543506	4.132892055	0.042057417	1
83857	ENSG00000133687	83857	TMTC1	-2.117916387	6.110997538	4.130759049	0.042110459	1
5284	ENSG00000162896	5284	PIGR	-4.663504245	8.157542863	4.129040499	0.042153245	1
5950	ENSG00000138207	5950	RBP4	-3.82700919	1.216178978	4.128899528	0.042156757	1
56956	ENSG00000143355	56956	LHX9	-3.948563173	-3.255286714	4.128486903	0.042167037	1
7846	ENSG00000167552	7846	TUBA1A	-2.361815478	9.165436867	4.127512523	0.042191325	1
340547	ENSG00000101842	340547	VSIG1	-6.322708411	5.566930093	4.126288061	0.042221867	1
4881	ENSG00000169418	4881	NPR1	1.724291033	6.053721035	4.122138408	0.042325546	1
347527	ENSG00000205667	347527	ARSH	-3.208437284	-1.298304817	4.116227733	0.042473686	1
10488	ENSG00000107175	10488	CREB3	-1.159327153	5.803342441	4.113890034	0.042532426	1
22822	ENSG00000139289	22822	PHLDA1	-2.120357661	5.79453471	4.113841744	0.042533641	1
115290	ENSG00000269190	115290	FBXO17	2.049767274	2.503832679	4.109868566	0.042633676	1
25769	ENSG00000155886	25769	SLC24A2	-4.233136913	-3.126529921	4.106475259	0.042719307	1
84502	ENSG00000092051	84502	JPH4	-6.316975473	0.110004006	4.105348996	0.042747768	1
80131	ENSG00000171017	80131	LRRC8E	1.414773734	4.197117197	4.104337818	0.042773338	1
219972	ENSG00000197629	219972	MPEG1	2.583096565	-0.404417149	4.103891614	0.042784627	1
9021	ENSG00000184557	9021	SOCS3	-1.551985118	4.187728916	4.101802772	0.042837514	1
57462	ENSG00000164976	57462	MYORG	-1.628774863	4.102208912	4.098891916	0.042911328	1
140766	ENSG00000138316	140766	ADAMTS14	-2.620815751	3.668365563	4.096658394	0.042968058	1
8814	ENSG00000100490	8814	CDKL1	1.896698125	1.593235559	4.091764576	0.043092631	1
7143	ENSG00000116147	7143	TNR	-7.273313833	-0.921521507	4.091371557	0.043102652	1
23092	ENSG00000145819	23092	ARHGAP26	1.350023566	6.805373931	4.090269725	0.043130759	1
89891	ENSG00000119333	89891	WDR34	-1.270070188	6.3136685	4.088682875	0.043171272	1
128710	ENSG00000149346	128710	SLX4IP	1.22248118	3.612953179	4.088200364	0.043183598	1
6531	ENSG00000142319	6531	SLC6A3	-5.31378626	-2.58999478	4.083924615	0.043292992	1
5322	ENSG00000127472	5322	PLA2G5	-4.174662653	-3.157278911	4.081526986	0.043354462	1
54674	ENSG00000173114	54674	LRRN3	-4.065699168	-1.840161922	4.081461506	0.043356142	1
3589	ENSG00000095752	3589	IL11	-4.162780147	-1.088472329	4.078411405	0.043434476	1
30008	ENSG00000172638	30008	EFEMP2	1.544615525	4.978135829	4.078097888	0.043442537	1
140876	ENSG00000042062	140876	RIPOR3	-3.473145607	2.722036078	4.077929328	0.043446871	1
4628	ENSG00000133026	4628	MYH10	1.460734394	8.822508657	4.077176278	0.04346624	1
80117	ENSG00000179674	80117	ARL14	-3.957689745	1.531395508	4.075961384	0.043497507	1
401285	ENSG00000166984	401285	TCP10L2	-4.906159756	-2.760432068	4.07559712	0.043506886	1
1293	ENSG00000163359	1293	COL6A3	-2.773638558	5.245522665	4.073427793	0.043562788	1
157680	ENSG00000132549	157680	VPS13B	1.086367675	7.446963966	4.070999423	0.043625455	1
3856	ENSG00000170421	3856	KRT8	-1.402805289	11.12915551	4.070063705	0.043649628	1
3791	ENSG00000128052	3791	KDR	-3.530305141	2.613418139	4.069740816	0.043657973	1
1551	ENSG00000160870	1551	CYP3A7	2.197700816	1.315656437	4.069487632	0.043664517	1
51655	ENSG00000108551	51655	RASD1	-2.02353906	2.999164817	4.068298033	0.04369528	1
1237	ENSG00000179934	1237	CCR8	-7.213908178	1.73293096	4.064998017	0.043780736	1
345275	ENSG00000170509	345275	HSD17B13	2.639896836	-2.743836957	4.064500001	0.043793648	1
4648	ENSG00000169994	4648	MYO7B	-2.10381508	2.788006943	4.062803695	0.043837658	1
10844	ENSG00000130640	10844	TUBGCP2	0.976933149	7.364776842	4.060958162	0.043885591	1
134864	ENSG00000146399	134864	TAAR1	-7.757293842	-0.517930747	4.058411994	0.043951813	1
91147	ENSG00000164953	91147	TMEM67	1.160988352	4.485349316	4.047031715	0.044249085	1
56245	ENSG00000205929	56245	C21orf62	-5.858773467	-2.118341903	4.04592165	0.044278195	1
105378663	ENSG00000228436	105378663	LOC105378663	-4.058071168	0.240669246	4.043307026	0.044346839	1

	Genes	Entrez	Symbol	logFC	logCPM	LR	PValue	FDR
651746	ENSG00000164236	651746	ANKRD33B	1.631179493	3.679931275	4.042178184	0.04437651	1
347252	ENSG00000137142	347252	IGFBPL1	-4.20674958	1.137152216	4.040972356	0.044408228	1
54461	ENSG00000159069	54461	FBXW5	-1.36004518	6.980270941	4.040450762	0.044421955	1
137695	ENSG00000167904	137695	TMEM68	1.085512146	4.636611763	4.040337367	0.04442494	1
654463	ENSG00000214814	654463	FER1L6	-3.550834787	3.237883246	4.036502472	0.044526011	1
60495	ENSG00000172987	60495	HPSE2	-7.796440125	-0.480164354	4.034640205	0.044575179	1
154043	ENSG00000153721	154043	CNKSR3	1.429036132	4.610043948	4.033106726	0.04461571	1
121227	ENSG00000139263	121227	LRIG3	1.307856862	5.811164289	4.031475883	0.044658856	1
57209	ENSG00000198105	57209	ZNF248	1.557574414	5.435508243	4.027378951	0.044767441	1
79652	ENSG00000131634	79652	TMEM204	-4.27742078	-3.107325654	4.026727488	0.044784733	1
55304	ENSG00000172296	55304	SPTLC3	1.551933724	4.361309008	4.02668353	0.0447859	1
8535	ENSG00000141582	8535	CBX4	-1.344364279	5.611000876	4.025393956	0.044820151	1
6779	ENSG00000126549	6779	STATH	-7.388627934	-0.929729967	4.021333643	0.044928173	1
81849	ENSG00000117069	81849	ST6GALNAC5	2.3563527	2.914428549	4.015989762	0.045070762	1
10103	ENSG00000117472	10103	TSPAN1	-2.170783353	7.171737422	4.012685776	0.045159159	1
2170	ENSG00000121769	2170	FABP3	-2.918640422	2.655032789	4.010871224	0.045207785	1
8406	ENSG00000101955	8406	SRPX	-3.747463833	2.365604437	4.009728567	0.045238433	1
23414	ENSG00000169946	23414	ZFPM2	2.314476798	4.626106056	4.009483888	0.045244999	1
1381	ENSG00000166426	1381	CRABP1	-5.639782412	3.165628548	4.003108065	0.045416442	1
63910	ENSG00000101194	63910	SLC17A9	2.533160985	2.303733711	3.996854625	0.045585258	1
57125	ENSG00000161381	57125	PLXDC1	-2.480801905	-1.995968302	3.994872601	0.045638903	1
80332	ENSG00000149451	80332	ADAM33	2.792571084	0.087575059	3.994795174	0.045640999	1
5343	ENSG00000183281	5343	PLGLB1	3.113317863	-3.078230531	3.990007651	0.045770856	1
2176	ENSG00000158169	2176	FANCC	-1.624888838	3.046649666	3.98822462	0.045819319	1
128611	ENSG00000124203	128611	ZNF831	-4.458579597	-3.012394242	3.987247495	0.0458459	1
90427	ENSG00000104081	90427	BMF	-2.218907819	3.056302227	3.986233844	0.045873492	1
7133	ENSG00000028137	7133	TNFRSF1B	-2.963711866	2.73820919	3.985740025	0.04588694	1
27348	ENSG00000136816	27348	TOR1B	-1.232975759	5.151293264	3.983763302	0.045940814	1
326624	ENSG00000172794	326624	RAB37	-3.411264872	-2.253137985	3.979841699	0.046047891	1
79827	ENSG00000166250	79827	CLMP	-4.185702504	0.403375304	3.978468362	0.046085451	1
5641	ENSG00000100600	5641	LGMN	-1.253639677	6.513327307	3.976932268	0.046127501	1
10982	ENSG00000166974	10982	MAPRE2	-2.478745814	3.218783518	3.974980222	0.046180996	1
79772	ENSG00000175471	79772	MCTP1	2.967624473	2.594752615	3.971794224	0.046268447	1
55016	ENSG00000145416	55016	MARCHF1	3.8199253	-0.117289269	3.969764239	0.046324258	1
696	ENSG00000124557	696	BTN1A1	-5.191679327	-2.546344329	3.96007998	0.04659149	1
168417	ENSG00000197123	168417	ZNF679	-7.026748587	-1.166784236	3.957275578	0.046669179	1
388743	ENSG00000203697	388743	CAPN8	-3.083383847	1.40052891	3.955981228	0.046705081	1
7448	ENSG00000109072	7448	VTN	-4.68273577	-0.616726132	3.950300009	0.046863012	1
4064	ENSG00000134061	4064	CD180	3.616183291	-3.349784323	3.949783735	0.046877392	1
1045	ENSG00000165556	1045	CDX2	-5.456714127	0.02058284	3.949259549	0.046891997	1
388650	ENSG00000154511	388650	DIPK1A	-2.070344207	3.127265329	3.947984766	0.046927535	1
595101	ENSG00000183604	595101	SMG1P5	1.703599145	0.518676768	3.946907749	0.046957581	1
100873998	ENSG00000230749	100873998	MEIS1-AS2	2.372000018	-1.970325937	3.944867958	0.047014543	1
945	ENSG00000105383	945	CD33	3.672937691	-2.771879978	3.943503566	0.047052685	1
2736	ENSG00000074047	2736	GLI2	-2.492197366	2.801668556	3.943184504	0.047061609	1
2719	ENSG00000147257	2719	GPC3	-3.076966011	1.204915574	3.942492355	0.047080975	1
246777	ENSG00000258484	246777	SPESP1	-7.085177535	-1.119024258	3.933340742	0.047337818	1
440699	ENSG00000162763	440699	LRRC52	-5.672473491	-2.24987644	3.931665842	0.047384984	1
54904	ENSG00000147548	54904	NSD3	0.943589119	7.933692987	3.925446798	0.047560551	1
51474	ENSG00000050405	51474	LIMA1	-1.457868617	7.0786817	3.925372774	0.047562645	1
374786	ENSG00000176927	374786	EFCAB5	2.053707016	0.482795171	3.924404562	0.047590041	1
128218	ENSG00000179178	128218	TMEM125	-2.236233769	3.391672251	3.92237071	0.047647644	1
651337	ENSG00000255585	651337	LOC651337	-3.695408318	-3.369151267	3.92069518	0.047695154	1
112637020	ENSG00000184068	112637020	SREBF2-AS1	-4.832793549	-2.871664889	3.917978425	0.047772294	1
9052	ENSG00000013588	9052	GPRC5A	-3.076605972	7.723650063	3.916009736	0.047828276	1
26085	ENSG00000167759	26085	KLK13	-4.165413096	3.556891346	3.913726354	0.047893293	1
9951	ENSG00000182601	9951	HS3ST4	4.543282103	-0.988752681	3.912892892	0.047917048	1
26153	ENSG00000066735	26153	KIF26A	-3.503117571	1.255253307	3.905737814	0.048121493	1
2139	ENSG00000064655	2139	EYA2	2.003241717	6.859337704	3.897171934	0.048367462	1
641298	ENSG00000237296	641298	SMG1P1	1.687411426	1.893017398	3.891954684	0.048517924	1

	Genes	Entrez	Symbol	logFC	logCPM	LR	PValue	FDR
11190	ENSG00000126001	11190	CEP250	1.172537377	6.708554765	3.886595322	0.048673	1
54847	ENSG00000072858	54847	SIDT1	-3.636801158	0.687228274	3.883797998	0.04875415	1
79022	ENSG00000134291	79022	TMEM106C	-1.32394407	6.523814997	3.881660257	0.048816261	1
5083	ENSG00000198807	5083	PAX9	-2.910497774	-1.548300674	3.88084658	0.048839924	1
133522	ENSG00000155846	133522	PPARGC1B	1.527556608	4.354206952	3.879793191	0.048870577	1
54436	ENSG00000125089	54436	SH3TC1	-2.841671662	5.069091612	3.874384106	0.049028295	1
1756	ENSG00000198947	1756	DMD	2.145636188	3.962706684	3.874082516	0.049037105	1
9779	ENSG00000131374	9779	TBC1D5	0.963664191	6.442580124	3.87320018	0.049062888	1
5156	ENSG00000134853	5156	PDGFRA	-3.591791115	1.784726987	3.872029719	0.049097112	1
1469	ENSG00000170373	1469	CST1	-5.350095497	6.49371478	3.869501813	0.049171115	1
85443	ENSG00000163673	85443	DCLK3	-3.843921105	-3.30461673	3.869482517	0.04917168	1
8844	ENSG00000141068	8844	KSR1	1.168214403	4.082044051	3.86630001	0.049265014	1
57495	ENSG00000174145	57495	NWD2	-5.827598786	-2.209768178	3.866165142	0.049268974	1
653677	ENSG00000232871	653677	SEC1P	2.909839801	-3.544543738	3.865176826	0.049297999	1
533	ENSG00000117410	533	ATP6V0B	-1.346362283	6.553308439	3.863668673	0.049342325	1
387804	ENSG00000214376	387804	VSTM5	-4.193666198	-1.670758243	3.859424635	0.049467289	1
136991	ENSG00000154438	136991	ASZ1	-3.909084704	-3.271169617	3.859015605	0.049479351	1
399	ENSG00000168421	399	RHOH	-4.145279827	-3.16788305	3.858171602	0.049504248	1
54716	ENSG00000163817	54716	SLC6A20	-2.988804229	6.966800784	3.855872654	0.049572133	1
3479	ENSG00000017427	3479	IGF1	-4.191302664	0.007500527	3.854051829	0.049625969	1
1514	ENSG00000135047	1514	CTSL	-1.347987369	5.662033774	3.853629298	0.049638471	1
200010	ENSG00000117834	200010	SLC5A9	-5.165256989	-2.686256684	3.850192893	0.04974027	1
2921	ENSG00000163734	2921	CXCL3	-1.994169214	4.999874628	3.847669696	0.049815157	1
23078	ENSG00000102763	23078	VWA8	0.970259047	6.223562511	3.842203209	0.049977808	1
5047	ENSG00000122133	5047	PAEP	-3.837349285	4.082737553	3.841992001	0.049984104	1

Supplemental Figure 1

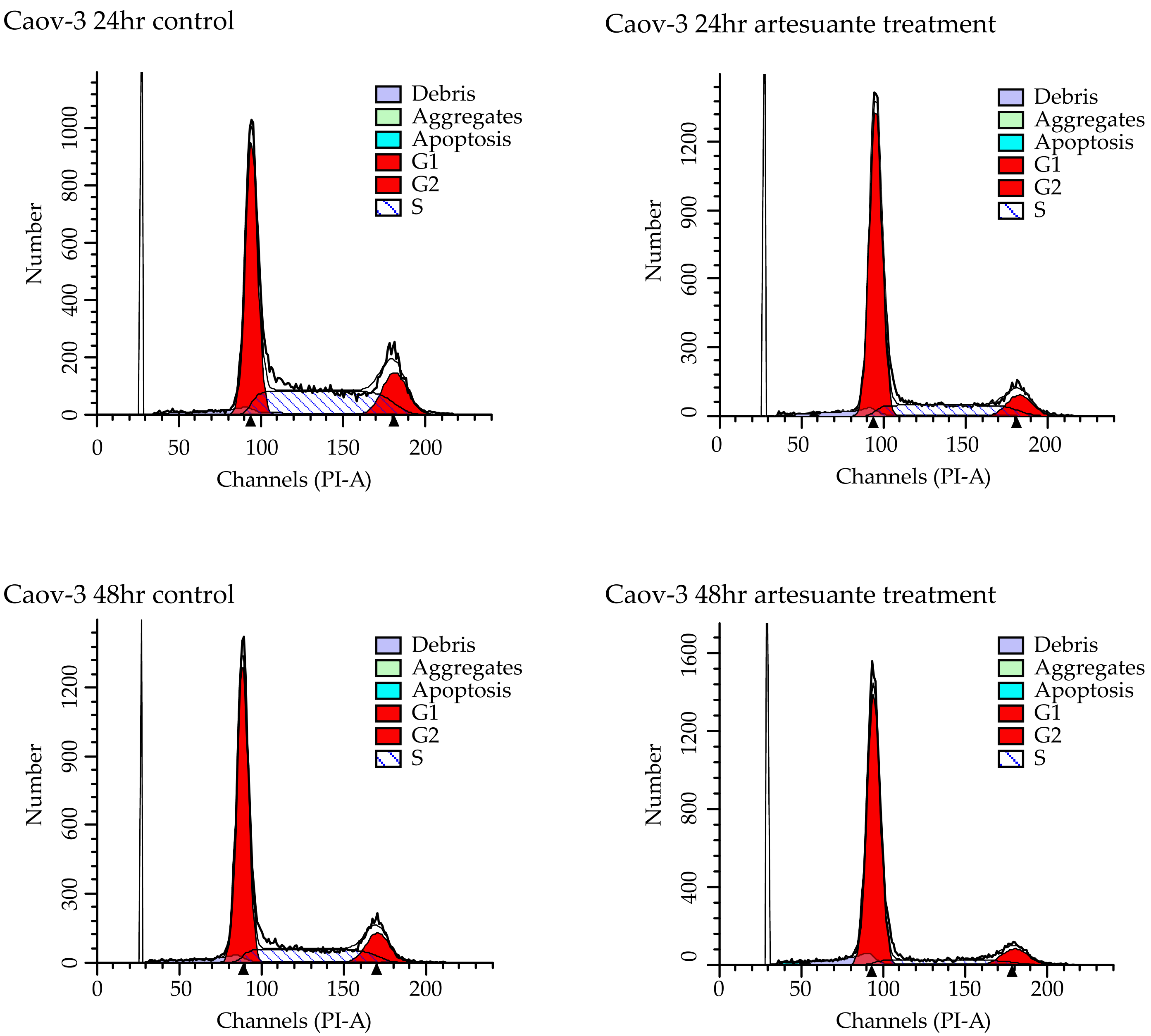

Supplemental figure 1: Representative flow cytometry histograms from a single experiment are provided for Caov-3 24 hr control and treatment and Caov-3 48 hr control and treatment. Raw data from replicate experiments are used in calculations. Each time point is treated with 0.1% DMSO (control) or 10μM artesunate. In Caov-3 cells, 48-hour artesunate treatment results in an increased percentage of cells in G1 78.98% +/- 0.9546 compared to 61.23% +/- 1.789 in vehicle-treated cells (P=0.0032). The percentage of cells in S-phase following 48 hour treatment with artesunate reveals a significant decrease in cells in S-phase, 12.14% +/- 0.1556 compared to 24.42% +/- 0.7354 in vehicle-treated cells (p<0.0009).

Supplemental Figure 2

UWB1 24hr control

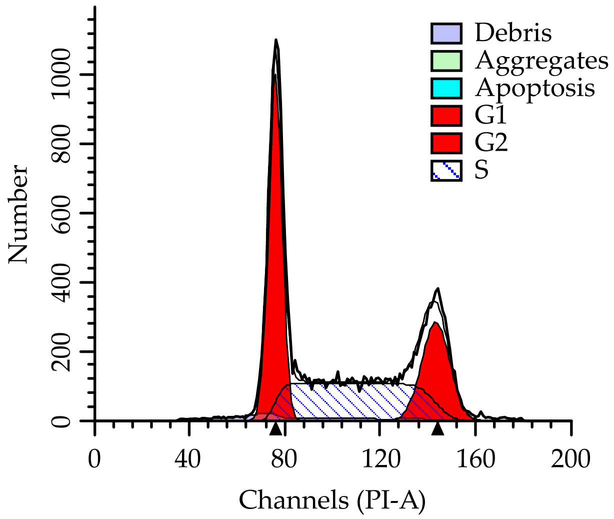

UWB1 24hr artesuante treatment

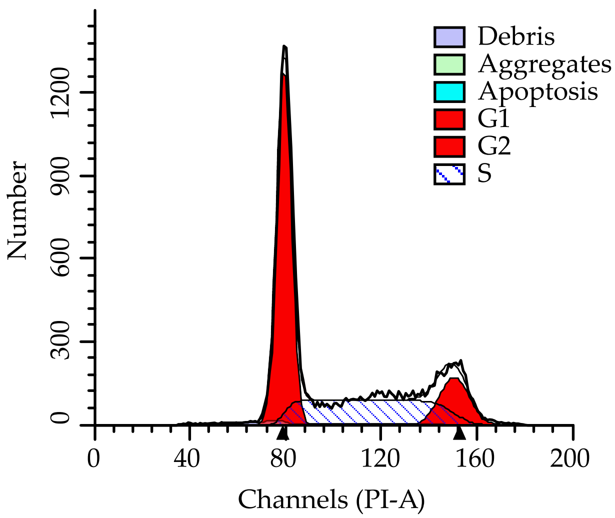

UWB1 48hr control

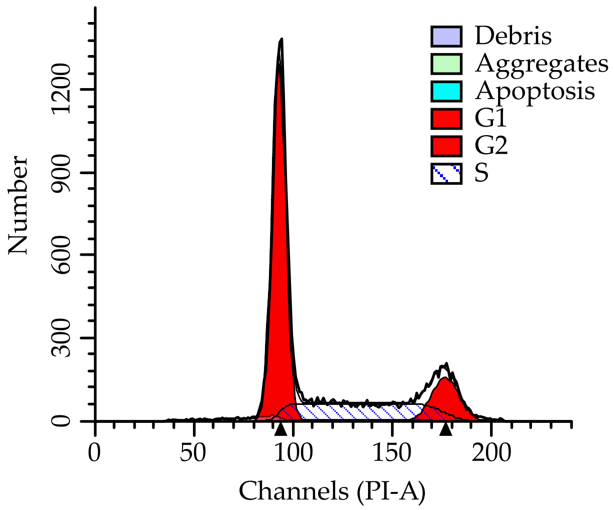

UWB1 48hr artesuante treatment

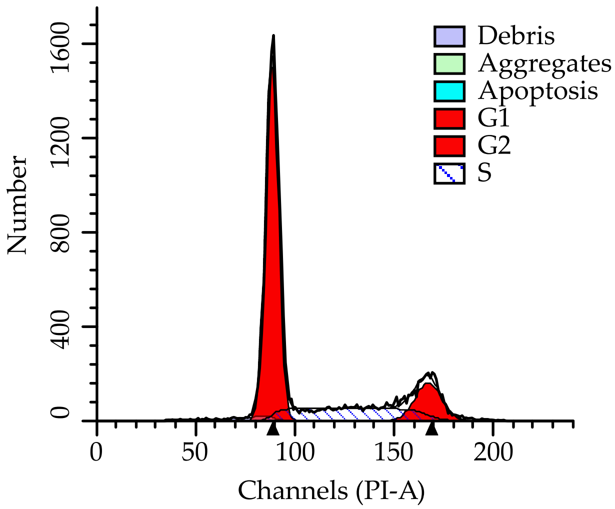

Supplemental figure 2: Representative flow cytometry histograms from a single experiment are provided for UWB1 24 hour control and treatment and UWB1 48 hr control and treatment. Raw data from replicate experiments are used in calculations. Each time point is treated with 0.1% DMSO (control) or 10μM artesunate. UWB1 cells had 65.35% +/- 0.0849 cells in G1 after artesunate treatment compared to 60.35% +/- 2.418 of control cells (p=0.0499). The percentage of cells in S-phase following 48 hour treatment with artesunate reveals a significant decrease in cells in S-phase, 18.87% +/- 2.779 compared to 26.25% +/- 2.234 of control cells (p<0.0497).
